# Supplementary material for: Early childhood risk factors for constipation and soiling at school age: an observational cohort study
Source: BMJ Paediatr Open. 2018 Feb 21;2(1):e000230. doi: 10.1136/bmjpo-2017-000230 (PMC5843013; doi:10.1136/bmjpo-2017-000230)
Supplement: Supplementary file 1 [file bmjpo-2017-000230supp001.pdf]

Table S1. Goodness of fit statistics for LLCA model of constipation

Sample	# classes	SSA-BIC	Entropy	LMR-p	BLRT-p
Cases with complete data for six measures of constipation (N=5,435)	1 class	21959.6	-	-	-
	2 classes	18334.3	0.878	<0.001	<0.001
	3 classes	18239.7	0.836	<0.001	<0.001
	4 classes	18209.0	0.842	<0.001	<0.001
	5 classes	18229.5	0.863	0.223	0.090
Cases with at least 3 non-missing measures of constipation (N=8,979)	1 class	32429.9	-	-	-
	2 classes	27293.9	0.859	<0.001	<0.001
	3 classes	27159.6	0.808	<0.001	<0.001
	4 classes	27078.8	0.818	<0.001	<0.001
	5 classes	27103.7	0.836	0.661	0.050
Cases with at least 1 non-missing measure of constipation (N=11,195)	1 class	34855.7	-	-	-
	2 classes	29567.9	0.813	<0.001	<0.001
	3 classes	29431.7	0.772	<0.001	<0.001
	4 classes	29342.3	0.785	<0.001	<0.001
	5 classes	29367.8	0.805	0.600	0.050

The LLCA model permits the inclusion of partial respondents (those who provide some but not all repeated measures) through the use of Full Information Maximum Likelihood (FIML) estimation. FIML is a commonly used approach for estimating models within the SEM framework and is often computationally less demanding than Multiple Imputation (MI). Both FIML and MI are based on the Missing At Random assumption. In the case of FIML, MAR equates to the notion that, conditional on the variables in the estimated model, there are no systematic differences between observed and non-observed values of any partially complete variable.

SSA-BIC - sample-size adjusted Bayesian Information Criterion. The Bayesian Information Criterion (BIC) is the traditional fit statistic for comparing mixture models. BIC will typically decrease and then increase following the incremental additional of classes. Using this statistic, the model with the lowest BIC (or other models with BIC values in that vicinity) would be deemed optimal. There is good support for 4-classes using the BIC.

Entropy. This is a measure of classification accuracy, and whilst it is generally of little use in determining the optimal model, it indicates the level of bias which one would expect were a standard three-step estimation to be performed. Here entropy is fairly high and above the commonly-used threshold of 0.8 for all models. We have recently demonstrated (Heron, Croudace, Barker, & Tilling, 2015) that bias can result even for values reaching 0.9 and hence we have opted for the bias-adjusted three-step method as described in the methods.

The *Bootstrap Likelihood Ratio Test (BLRT)* and the *Lo-Mendell-Rubin (LMR)* test statistics both assess change in model fit when adding an additional class. Here a high p-value for a k-class model indicates no substantial improvement in fit compared to the k-1 class solution. Whilst the BLRT is usually more conservative, both are in support of a 4-class solution.

Heron, J. E., Croudace, T. J., Barker, E. D., & Tilling, K. (2015). A comparison of approaches for assessing covariate effects in latent class analysis. Longitudinal and Life Course Studies; Vol 6, No 4 (2015): Longitudinal and Life Course Studies.

Table S2. Goodness of fit statistics of parallel LLCA models including between 3 and 5 classes both on soiling and constipation dimension.

# soiling classes	# constipation classes	Complete data for both soiling *and* constipation (N=4,931)		At least 3 measures each for both soiling *and* constipation (N=8,435)		At least 1 measure each for both soiling *and* constipation (N=10,450)	
		Entropy	SSA-BIC	Entropy	SSA-BIC	Entropy	SSA-BIC
3	3	0.833	25598.3	0.823	40364.7	0.801	44632.6
3	4	0.843	25574.4	0.832	40298.0	0.810	44563.6
3	5	0.847	25602.4	0.846	40328.6	0.826	44596.9
4	3	0.859	25594.6	0.846	40360.2	0.825	44628.7
4	4	0.879	**25571.9**	0.852	**40285.3**	0.830	**44552.1**
4	5	0.882	25603.4	0.866	40317.3	0.842	44586.0
5	3	0.841	25620.5	0.834	40393.9	0.794	44664.7
5	4	0.884	25597.3	0.870	40321.8	0.850	44589.9
5	5	0.893	25631.1	0.884	40357.0	0.861	44627.6

There was good support for a 4-class solution for both constipation and soiling when considered separately. When both sets of data were brought together for the estimation of a joint model we considered 3-, 4- and 5-class solutions for both dimensions. The reason being that the additional information can distort the estimation of the two latent class measurement models, particularly when missing data is present. In the current situation, there remained good support for a 4-class by 4-class solution and the individual trajectories estimated for soiling and constipation were not adversely affected.

Table S3. Association between risk factors and the latent classes of (i) Constipation, and (ii) Soiling

	(i) Constipation classes (n ≤ 8,979)				(ii) Soiling classes (n ≤ 8,764)			
	Early Childhood Occurrence OR [95% CI]	Late Childhood Occurrence OR [95% CI]	Persistent OR [95% CI]	Omnibus p-value	Delayed OR [95% CI]	Relapse OR [95% CI]	Persistent OR [95% CI]	Omnibus p-value
Hard stools regularity at 2.5 yrs				<0.001				0.146
Never hard (ref.)	1.00 ref				1.00 ref			
Sometimes	2.28 [1.32, 3.92]	1.47 [0.99, 2.20]	1.88 [1.11, 3.18]		1.00 [0.55, 1.81]	1.27 [0.75, 2.15]	0.79 [0.52, 1.19]	
Usually hard	2.24 [1.26, 3.98]	1.30 [0.83, 2.03]	2.19 [1.27, 3.78]		0.84 [0.43, 1.64]	0.74 [0.39, 1.40]	0.73 [0.46, 1.17]	
Breastfeeding by 6 months				0.121				0.588
child never breastfed (ref.)	1.00 ref				1.00 ref			
child breastfed for < 6mn	1.03 [0.69, 1.54]	1.46 [0.98, 2.16]	1.05 [0.71, 1.55]		1.30 [0.73, 2.32]	1.52 [0.87, 2.68]	0.85 [0.56, 1.28]	
child breastfed for at least 6mn	1.35 [0.90, 2.00]	1.39 [0.92, 2.10]	0.75 [0.48, 1.19]		1.12 [0.60, 2.09]	1.30 [0.71, 2.38]	0.99 [0.65, 1.50]	
Sex				<.001				<.001
Female (ref.)	1.00 ref				1.00 ref			
Male	0.81 [0.61 1.07]	0.64 [0.49, 0.84]	0.84 [0.62, 1.13]		1.45 [0.95, 2.22]	1.91 [1.30, 2.80]	2.00 [1.42, 2.83]	
Parental social class				0.066				0.721
Non-manual (ref)	1.00 ref				1.00 ref			
Manual	0.81 [0.52, 1.25]	0.68 [0.44, 1.05]	1.43 [0.98, 2.07]		0.76 [0.38, 1.55]	1.19 [0.74, 1.91]	1.16 [0.74, 1.80]	
Maternal educational attainment				0.121				0.795
A-level/degree (ref.)	1.00 ref				1.00 ref			
O-level	0.84 [0.60, 1.17]	0.88 [0.65, 1.18]	1.32 [0.93, 1.89]		1.07 [0.67, 1.70]	0.84 [0.55, 1.30]	0.91 [0.62, 1.32]	
< O-level	0.82 [0.57, 1.19]	0.64 [0.44, 0.94]	1.22 [0.82, 1.82]		0.67 [0.35, 1.28]	1.07 [0.69, 1.66]	1.00 [0.66, 1.51]	
Material hardship at 33 months				0.144				0.087
no hardship [score<5] (ref.)	1.00 ref				1.00 ref			
material hardship [score ≥ 5]	1.18 [0.86, 1.63]	1.00 [0.73, 1.37]	1.38 [0.99, 1.92]		0.83 [0.48, 1.42]	1.11 [0.74, 1.68]	1.53 [1.07, 2.18]	

Table S3. Continued

	(i) Constipation class				(ii) Soiling class			
	Early Childhood Occurrence OR [95% CI]	Late Childhood Occurrence OR [95% CI]	Persistent OR [95% CI]	Omnibus p-value	Delayed OR [95% CI]	Relapse OR [95% CI]	Persistent OR [95% CI]	Omnibus p-value
Home ownership at 33 months				0.022				0.791
Home owned/mortgaged (ref.)	1.00 ref				1.00 ref			
Privately rented	0.89 [0.42, 1.86]	0.58 [0.24, 1.41]	0.97 [0.42, 2.21]		1.05 [0.35, 3.16]	1.27 [0.56, 2.88]	0.89 [0.34, 2.35]	
Subsidised rented	0.77 [0.46, 1.28]	0.47 [0.25, 0.90]	1.73 [1.17, 2.57]		0.88 [0.43, 1.80]	0.56 [0.24, 1.28]	1.14 [0.69,1.88]	
Car ownership at 33 months				0.510				0.057
yes, car owned (ref.)	1.00 ref				1.00 ref			
no car	1.28 [0.79, 2.07]	0.65 [0.33, 1.26]	1.16 [0.67, 2.01]		1.32 [0.63, 2.77]	0.89 [0.40, 1.97]	1.90 [1.16, 3.09]	
Gestation age at delivery				0.248				0.309
≥ 37weeks (ref.)	1.00 ref				1.00 ref			
< 37 weeks	0.97 [0.84, 1.11]	0.49 [0.21, 1.14]	0.79 [0.39, 1.60]		1.49 [0.67, 3.27]	1.12 [0.50, 2.51]	1.50 [0.81, 2.77]	
Birth weight				0.075				0.467
≥ 2500g (ref.)	1.00 ref				1.00 ref			
< 2500g	1.13 [0.84, 1.53]	0.84 [0.72, 0.99]	1.38 [0.80, 2.37]		0.73 [0.21, 2.60]	1.23 [0.56, 2.68]	1.56 [0.83, 2.94]	
Developmental level at 18m				0.189	1.00 ref			<0.001
Per 1 SD reduction in development	0.97 [0.81, 1.18]	0.91 [0.77, 1.07]	1.21 [1.01, 1.46]		1.27 [1.04, 1.54]	1.31 [1.05, 1.63]	1.66 [1.39, 2.00]	
Toilet training initiation				0.991				0.012
Before 6mn	1.31 [0.52, 3.29]	1.16 [0.47, 2.85]	1.21 [0.44, 3.35]		1.91 [0.54, 6.78]	2.01 [0.78, 5.15]	1.13 [0.30, 4.30]	
Between 6 &15mn	1.01 [0.63, 1.59]	1.10 [0.74, 1.64]	1.00 [0.61, 1.62]		1.26 [0.66, 2.42]	0.70 [0.35, 1.41]	0.99 [0.56, 1.74]	
Started between 15 & 24mn (ref.)	1.00 ref				1.00 ref			
Started after 24mn	1.13 [0.82, 1.55]	0.90 [0.66, 1.24]	0.99 [0.70, 1.41]		1.60 [0.99, 2.58]	1.15 [0.76, 1.74]	1.62 [1.12, 2.33]	

Figure S1. Derivation of samples used for analysis

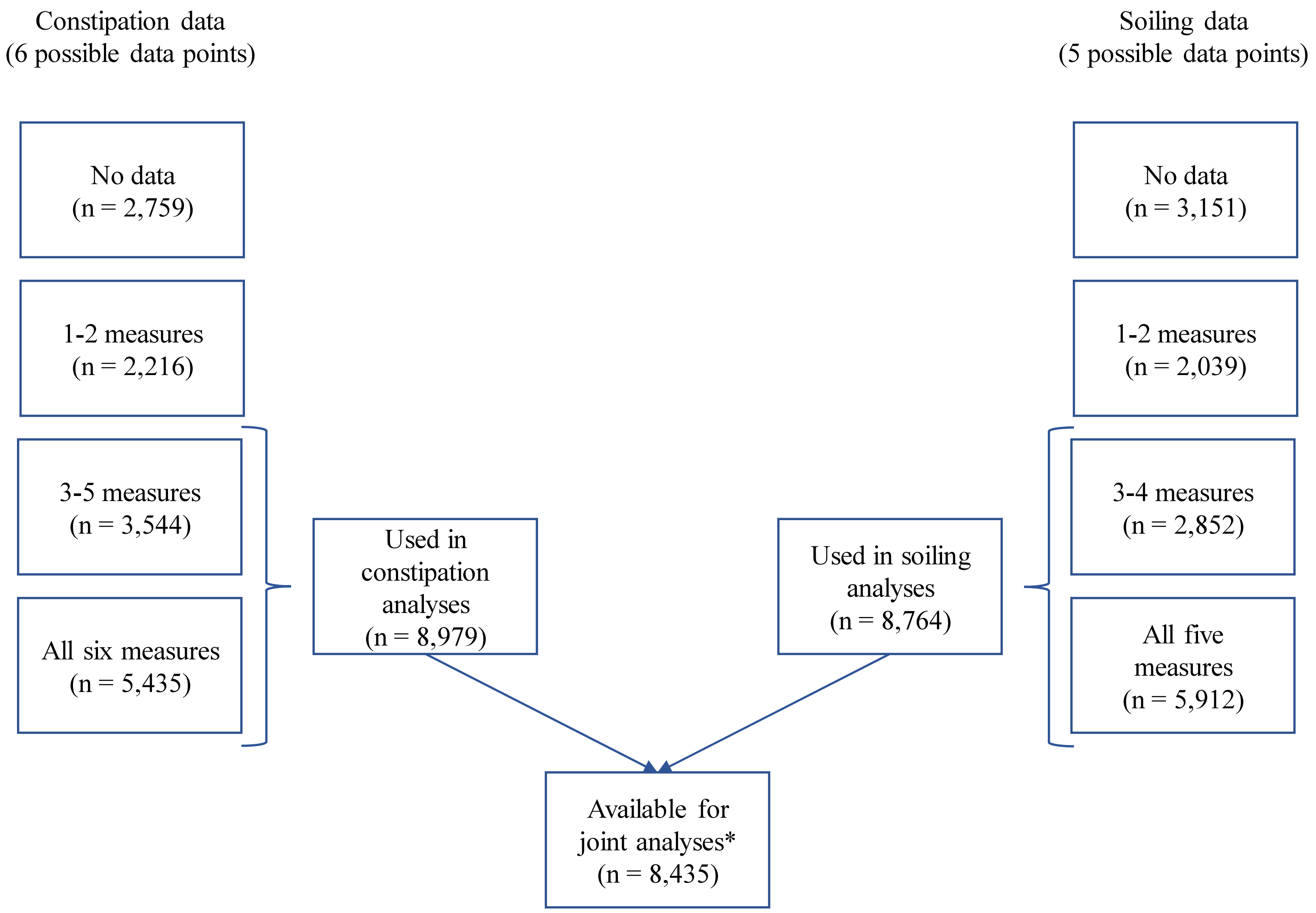

* Joint analysis focussed on the sample with 3+ measures on both constipation and soiling

Figure S2. Rates of daytime wetting, bedwetting, stomach ache and infrequent bowel movements within classes of children defined by their constipation and daytime soiling in mid-childhood.

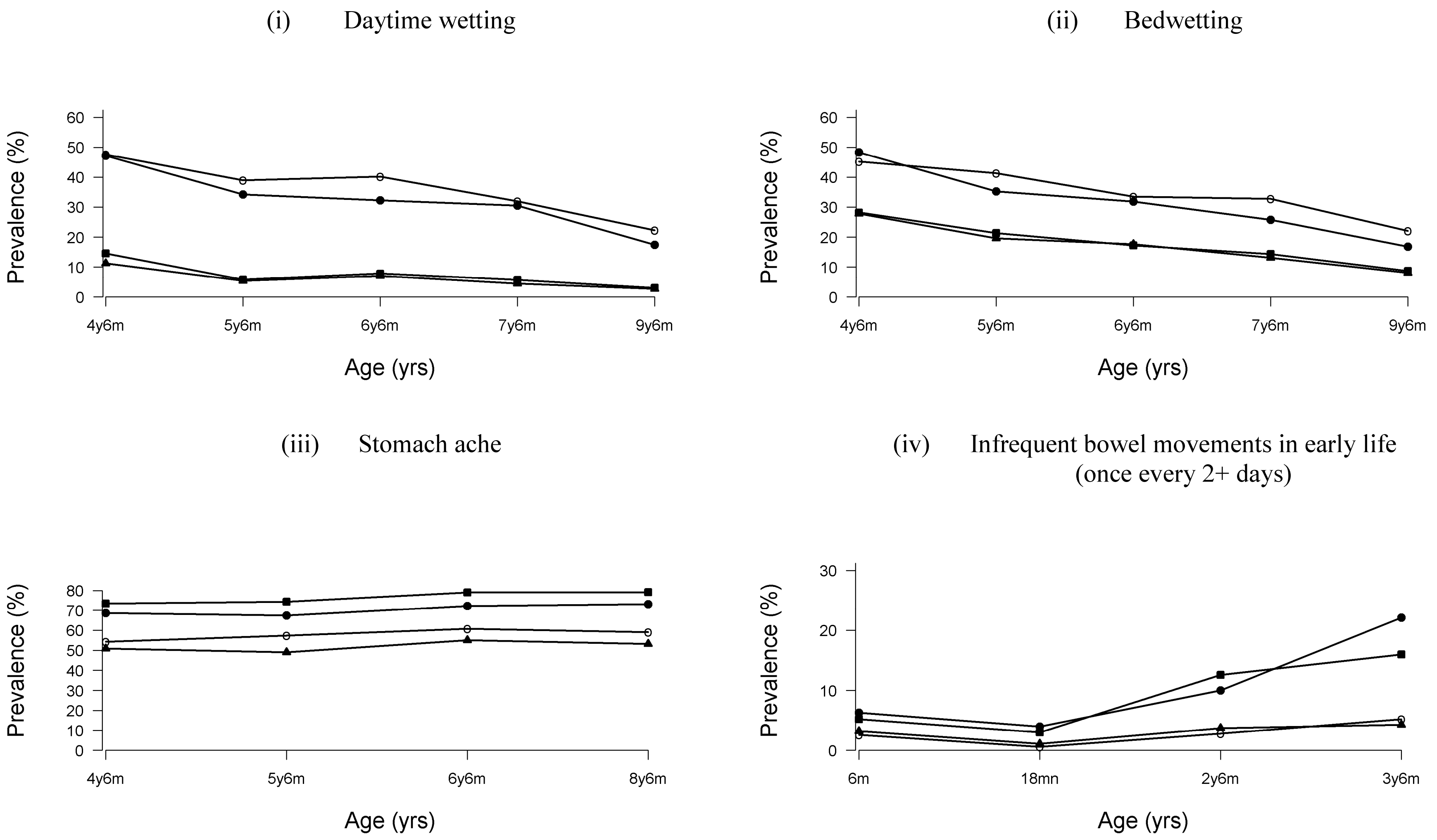

Constipation with soiling (●), Soiling alone (O), Constipation alone (■), Normative (▲)
