# Supplementary material for: Early childhood risk factors for constipation and soiling at school age: an observational cohort study
Source: BMJ Paediatr Open. 2018 Feb 21;2(1):e000230. doi: 10.1136/bmjpo-2017-000230 (PMC5843013; doi:10.1136/bmjpo-2017-000230)
Supplement: Author's manuscript [file bmjpo-2017-000230.draft_revisions.pdf]

BMJ Paediatrics Open

BMJ Paediatrics Open is committed to open peer review. As part of this commitment we make the peer review history of every article we publish publicly available.

When an article is published we post the peer reviewers' comments and the authors' responses online. We also post the versions of the paper that were used during peer review. These are the versions that the peer review comments apply to.

The versions of the paper that follow are the versions that were submitted during the peer review process. They are not the versions of record or the final published versions. They should not be cited or distributed as the published version of this manuscript.

BMJ Paediatrics Open is an open access journal and the full, final, typeset and author-corrected version of record of the manuscript is available on our site with no access controls, subscription charges or pay-per-view fees (http://bmjpaedsopen.bmj.com).

If you have any questions on BMJ Paediatrics Open's open peer review process please email info.bmjpo@bmj.com

BMJ Paediatrics Open

Early childhood risk factors for constipation and soiling at school age: an observational cohort study

Journal:	*BMJ Paediatrics Open*
Manuscript ID	bmjpo-2017-000230
Article Type:	Original article
Date Submitted by the Author:	31-Oct-2017
Complete List of Authors:	Heron, Jon; University of Bristol, Population Health Sciences<br>Grzeda, Mariusz; University of Bristol, Population Health Sciences<br>Tappin, David; University of Glasgow, Scottish Cot Death Trust, Child Health, School of Medicine<br>von Gontard, Alexander; Saarland University Hospital, Department of Child and Adolescent Psychiatry<br>Joinson, Carol; University of Bristol, Population Health Sciences
Keywords:	Gastroenterology

SCHOLARONE™
Manuscripts

Confidential: For Review Only

Early childhood risk factors for constipation and soiling at school age: an observational cohort study

Jon Heron[1], Mariusz Grzeda[1], David Tappin[2], Alexander von Gontard[3], Carol Joinson[1]

[1] Department of Population Health Sciences, Bristol Medical School, University of Bristol, UK.

[2] Scottish Cot Death Trust, Child Health, School of Medicine, University of Glasgow, UK.

[3] Department of Child and Adolescent Psychiatry, Saarland University Hospital, Homburg, Germany.

Corresponding author

Dr Jon Heron

Population Health Sciences, Bristol Medical School, University of Bristol

Oakfield House, Oakfield Grove, Bristol, BS8 2BN

Tel: 0117 3310093

Email: jon.heron@bristol.ac.uk

Word count: 2,797 words

Keywords: ALSPAC.

It is a requirement of the data providers that we include ALSPAC as a keyword however this was not permitted by the online submission system.

Confidential: For Review Only

A. What is known about the subject

Constipation and soiling are common in childhood, but little is known about factors that increase the risk of these problems persisting at primary school age.

The majority of earlier studies examining risk factors for childhood constipation and soiling are cross-sectional and many are based on small / clinic samples.

No prospective cohort studies have examined the association between risk factors in early childhood and different trajectories of constipation and soiling at primary school age.

B. What this study adds

This study finds evidence that risk factors in early childhood are differentially associated with different trajectories of childhood constipation and soiling.

Contrary to common beliefs of clinicians, we found that among children with soiling, those with soiling alone (i.e. without constipation) outnumbered those with associated constipation.

Experiencing hard stools in early childhood is a risk factor for constipation; developmental delay is a risk factor for soiling alone and constipation with soiling.

Confidential: For Review Only

ABSTRACT

Objective. Constipation and soiling are common in childhood. This study examines the co-morbidity between childhood constipation and soiling and early childhood risk factors for these problems.

Design. The sample comprised 8,979 participants from the Avon Longitudinal Study of Parents and Children with maternally-reported measures of constipation (6 time-points between 4–10yrs) and soiling (5 time-points between 4–9yrs). We used latent class analysis to extract longitudinal patterns of constipation and soiling. We examined whether the latent classes are differentially associated with maternally reported risk factors in early childhood (stool consistency, breastfeeding, socioeconomic background, gestation, birthweight, developmental level and age at initiation of toilet training) using multinomial logistic regression models.

Results. We extracted 4 latent classes: 'normative' (74·5%: very low probability of constipation or soiling); 'constipation alone' (13·2%); 'soiling alone' (7·5%) and 'constipation with soiling' (4·8%). Hard stools at 2½ years were associated with increased odds of constipation alone (1·90 [1·40, 2·60], reference= normative class). Developmental delay at 18 months was associated soiling alone (1·44 [1·28, 1·62]) and constipation with soiling (1·31 [1·13, 1·51]), but not constipation alone. We found limited evidence of associations with socioeconomic background and no evidence of associations with age at initiation of toilet training, breastfeeding, gestational age or birthweight.

Conclusion. Constipation alone was the most prevalent pattern in this cohort. Treatment for hard stools in early childhood is needed to prevent chronic constipation at school age. Constipation

Confidential: For Review Only

with soiling was less common than soiling alone. Further research is needed into the causes of non-retentive soiling.

Confidential: For Review Only

INTRODUCTION

Bowel problems are common in childhood and have a considerable impact on quality of life.[1] It is believed that 80% of faecal incontinence is due to overflow from chronic constipation, while 20% have no constipation (functional non-retentive fecal incontinence).[2] The Rome-IV definition for functional constipation at developmental age 4 years and above requires at least two of six symptoms (2 or fewer defecations in the toilet per week; at least 1 episode of fecal incontinence per week; history of retentive posturing/stool retention; history of painful or hard stools; a large fecal mass in the rectum; large diameter stools that can obstruct the toilet) present once a week or more for at least one month.[3] Rome-IV diagnostic criteria are also available for functional constipation in children under 4 years.[4] The diagnostic criteria for functional non-retentive faecal incontinence are: inappropriate defecation; no medical condition for symptoms, and no retention (criteria should be met for at least one month). [3] Other clinical definitions are sometimes used.[5,6] Epidemiological studies of the prevalence of constipation and soiling vary probably because of different definitions. A systematic review reported the median prevalence of constipation in 0–18-year-olds to be 8·9%, with similar prevalence in boys and girls,[5] however, more recent findings suggest a higher proportion of constipation in girls.[6] The prevalence of childhood soiling is between 1% and 4% and is consistently found to be two to four times more common in boys.[7,8] A recent large cross-sectional study of 5-13-year-olds reported that 7·8% (9·8% boys, 5·8% girls) experienced faecal incontinence.[9] Only one epidemiological survey, of 10–16-year-olds in Sri Lanka, differentiated between soiling with and without constipation and reported that 2·0% experienced faecal incontinence and 18% of those did not have constipation.[10]

Confidential: For Review Only

Early identification of children at risk of constipation and soiling could lead to timely interventions to reduce the adverse impacts on quality of life and psychosocial development. Clinicians believe that pain of passing hard stools in infancy and early childhood is the principal contributing factor for acute childhood constipation[11] leading to chronic constipation which causes soiling.[11,12] Hard stools lead to withholding and toileting refusal,[13] retaining a stool mass and increasing the difficulty of evacuating. Breastfed infants produce softer stools[14] and those breastfed for less than 6 months may develop constipation more commonly.[15] Other risk factors include lower levels of parental education,[9,16] income[9] and socioeconomic status,[8,10] low birthweight and prematurity[17] and developmental delay.[18] Timing of toilet training has also been investigated but findings are inconsistent.[7,19,20] Only one study specified whether constipation occurred with or without soiling.[11] Very little is known about risk factors for soiling without constipation. Finally, most earlier studies of risk factors for constipation and soiling are cross-sectional which makes the timing of events more difficult to determine.

Although most children achieve bowel control by 3–5 years,[19,21] there is recent evidence for different patterns of development of bowel control.[22] These ‘developmental trajectories’ distinguish children with normative development (89·0%), delayed attainment (4·1%), persistent soiling (2·7%) and relapses in soiling (4·1%).[22] Describing developmental trajectories of soiling alone does not allow the determination of whether soiling is occurring with or without constipation. The aims of this paper are twofold: First, we extend previous work using data from the ALSPAC birth cohort to determine the degree of co-morbidity between constipation and soiling in childhood and second, we examine the association between risk factors in early childhood and trajectories of constipation and soiling at primary school age.

Confidential: For Review Only

METHODS

Participants

The sample comprised participants from the Avon Longitudinal Study of Parents and Children (ALSPAC). Detailed information is available at (http://www.bristol.ac.uk/alspac), including a fully searchable data-dictionary (http://www.bris.ac.uk/alspac/researchers/data-access/data-dictionary). Pregnant women resident in the former Avon Health Authority in SW England, having an estimated date of delivery between 1/4/91 and 31/12/92 were invited to take part, resulting in a cohort of 14,541 pregnancies.[23] Of the 13,978 singletons/twins alive at one year, 24 participants withdrew consent leaving a starting sample of 13,954. Ethical approval was obtained from the ALSPAC Law and Ethics committee and local research ethics committees.

Soiling and constipation in mid-childhood

When children were 4½, 5½, 6½, 7½ and 9½ years their parents were asked: "How often usually does your child dirty his/her pants during the day?" with options: 'Never'; 'Occasional accident but less than once/week'; 'About once/week'; '2-5 times/week'; 'Nearly every day'; and 'More than once a day'. Responses were collapsed to indicate presence of soiling at each age. Parents were also asked about their child's constipation across a similar age range: "Has he/she had any constipation in the past 12 months?" with options: 'Yes, and saw a doctor'; 'Yes, but did not see the doctor', and 'No, did not have'. Responses were collapsed to indicate presence of constipation at each age.

Risk factors in early childhood

Potential risk factors were identified from the literature. Data were obtained from questionnaires completed by mothers and included the child's stool consistency at 2½ years, breastfeeding during the first six months, family socioeconomic position assessed during the antenatal period

Confidential: For Review Only

(parental social class, maternal educational attainment) or at 33 months (material hardship, home ownership and car access), length of gestation and birthweight, developmental level[24] and age at initiation of toilet training (assessed when the child was 6, 15, and 24 months).

Statistical modelling

We have previously used longitudinal Latent Class Analysis (LLCA) to derive developmental trajectories of soiling[22] where we showed that patterns of soiling at 4–9 years could be adequately explained by a four-class solution. However, such a model ignores the comorbidity with constipation. Therefore, in the current study we (i) estimated a similar latent class model of for constipation and (ii) combined this with soiling in a parallel model to describe changes in both problems (using Mplus v7·11[25]). Further details can be found in the supplementary materials. Once the optimal model had been established, we examined the association between the risks (above) and constipation/soiling class-membership using multinomial logistic regression. Coefficients and standard errors were estimated using the bias-adjusted three-step approach[26,27] which has been shown to reduce the bias inherent in such regression models.[26] For most risks we report univariable estimates of association, however for toilet training initiation we considered the potential confounding effects of gender, social class, early parenthood, mother’s education, housing adequacy, major financial difficulties, family size, social network/support, developmental delay, and maternal depression.

Confidential: For Review Only

RESULTS

Table 1 shows rates of soiling and constipation at each age. Whilst rates of reported constipation decrease steadily, there is a more complex pattern for soiling. Of those reporting constipation, the proportion who saw a doctor decreased from 19·7% to 11·6% (data not shown).

Table 1. Prevalence of soiling and constipation in the ALSPAC study

Subset of dataset containing at least one non-missing time points for both soiling and constipation (max N = 10,450)

	4½ years	5½ years	6½ years	7½ years	9½ years	
No soiling	8720 (92.8%)	8284 (93.8%)	7752 (92.2%)	7617 (93.1%)	7288 (94.8%)	
Soiling	673 (7.2%)	548 (6.2%)	654 (7.8%)	565 (6.9%)	397 (5.2%)	
	4years 9mn	5years 9mn	6years 9mn	7years 7mn	8years 7mn	10years 8mn
No constipation	7947 (85.6%)	7622 (89.6%)	7580 (89.7%)	7346 (89.9%)	7062 (90.2%)	6631 (90.4%)
Constipation	1342 (14.4%)	881 (10.4%)	873 (10.3%)	822 (10.1%)	765 (9.8%)	707 (9.6%)

Subset of dataset containing at least three non-missing time points for both soiling and constipation, (max N=8,435)

	4½ years	5½ years	6½ years	7½ years	9½ years	
No soiling	7,507 (92.8%)	7,518 (94.0%)	7,268 (92.2%)	7,153 (93.2%)	6,801 (94.8%)	
Soiling	585 (7.2%)	477 (6.0%)	611 (7.8%)	525 (6.8%)	372 (5.2%)	
	4years 9mn	5years 9mn	6years 9mn	7years 7mn	8years 7mn	10years 8mn
No constipation	6,893 (85.5%)	7,014 (89.7%)	7,086 (89.6%)	6,894(90.0%)	6,549 (90.5%)	6,194 (90.6%)
Constipation	1,173 (14.5%)	809 (10.3%)	825 (10.4%)	766 (10.0%)	689 (9.5%)	644 (9.4%)

Sample with complete data on all eleven measures, (N=4,931)

	4½ years	5½ years	6½ years	7½ years	9½ years	
No soiling	4575 (92.8%)	4638 (94.1%)	4568 (92.6%)	4590 (93.1%)	4670 (94.7%)	
Soiling	356 (7.2%)	293 (5.9%)	363 (7.4%)	341 (6.9%)	261 (5.3%)	
	4years 9mn	5years 9mn	6years 9mn	7years 7mn	8years 7mn	10years 8mn
No constipation	4244 (86.1%)	4425 (89.7%)	4402 (89.3%)	4426 (89.8%)	4454 (90.3%)	4458 (90.4%)
Constipation	687 (13.9%)	506 (10.3%)	529 (10.7%)	505 (10.2%)	477 (9.7%)	473 (9.6%)

Confidential: For Review Only

Unconditional model for constipation

There was good support for a 4-class solution when considering the six repeated measurements of constipation. Focussing on the sample consisting of cases with at least 3 valid/non-missing measures (n=8,979) the following classification of children was obtained: Normative (82%; very low probability of constipation throughout), Early childhood occurrence (7%; children suffering from constipation until 6 years), Late childhood occurrence (8% problem with constipating emerging after 6 years), Persistent (3%; high probability of problems throughout).

Parallel model for soiling and constipation

The next step was to merge the two LLCA models into a single parallel model permitting an investigation into the longitudinal association between soiling and constipation- see **Table 2** and **Figure 1**. Based on these results we collapsed the 16 groups defined by such a two-way classification into four clinical relevant subgroups to facilitate further study. The four resulting groups were: *Normative* (74·5% of total) - Normative classes for both constipation and soiling; *constipation alone* (13·2%) - Normative class for soiling and non-normative classes for constipation; *soiling alone* (7·5%) - Normative class for constipation and non-normative classes for soiling; *constipation with soiling* (4·8%) – classified as non-normative in terms of the progression of both constipation and soiling during the period of childhood studied.

Confidential: For Review Only

Table 2. Associations between constipation and soiling: Joint distribution of classes derived from parallel LLCA model (n=8,435).

	Soiling classes				
**Constipation classes**	Normative (soiling)	Delayed	Relapse	Persistent	Total
Normative (constipation)	74.5%[a]	4.3%[c]	2.2%[c]	1.0%[c]	82%
Early childhood occurrence	5.5%[b]	1.1%[d]	<0.1%[d]	0.5%[d]	7.1%
Late childhood occurrence	5.8%[b]	0.6%[d]	0.9%[d]	0.3%[d]	7.6%
Persistent	1.9%[b]	0.4%[d]	0.5%[d]	0.5%[d]	3.3%
Total	87.7%	6.4%	3.6%	2.3%	100%

Footnote:

Data shown in the table are class proportions for the latent class patterns based on the estimated model. These results are for the sample of children with both constipation and soiling data for at least three time points.

[a] Normative (74.5% of total) - Normative classes for both constipation and soiling

[b] Constipation alone (13.2% of total) - Normative class for soiling and non-normative classes for constipation

[c] Soiling alone (7.5% of total) - Normative class for constipation and non-normative classes for soiling

[d] Constipation with soiling (4.8% of total) - Non-normative classes for constipation and soiling

Confidential: For Review Only

Rates of associated symptoms within each class

To gain further insight into this classification we examined the extent to which the rates of daytime wetting, bedwetting and stomach ache in mid-childhood, and infrequent bowel movements in early-childhood differed across the four composite classes (Figure S1). Firstly, the two classes involving soiling exhibited similar rates of both daytime wetting and bedwetting and furthermore these rates were consistently higher than the constipation only and the normative class. Secondly, rates of stomach ache were lower for the soiling alone class compared to either class reporting constipation. Finally, in terms of the rates of infrequent bowel movements in early-childhood, those classified as soiling alone were no different to the normative class for whom infrequent movements were rare, whereas both classes experiencing constipation were seen to have infrequent bowel movements which became a more common finding as the children became older.

Risk factors for Constipation / Soiling class membership

Table 3 shows the association between the risk factors and odds-ratios for membership of the classes of 'constipation alone', 'soiling alone' and 'constipation with soiling' with reference to the normative class. For completeness, we append the results for the separate models of soiling and constipation (Table S3).

Confidential: For Review Only

Table 3. Association between risk factors and the parallel classes of soiling and constipation (n ≤ 8,435 depending on risk factor)

	n (%)	Constipation alone OR [95% CI]	Soiling alone OR [95% CI]	Constipation with soiling OR [95% CI]	Omnibus p-value
Sex					
Female (ref)	4,082 (52%)				
Male	4,353 (48%)	0.63 [0.53, 0.75]	1.78 [1.39, 2.27]	1.38 [1.06, 1.79]	<0.001
Hard stools regularity at 2½ years					
Never hard (ref)	1,414 (18%)				
Sometimes	4,312 (54%)	2.08 [1.56, 2.77]	1.12 [0.82, 1.52]	1.22 [0.84, 1.78]	<0.001
Usually hard	2,235 (28%)	1.90 [1.40, 2.60]	0.68 [0.46, 0.99]	1.26 [0.85, 1.89]	
Breastfeeding					
Child never breastfed (ref)	1,695 (21%)				
Child breastfed for < 6 months	3,579 (45%)	1.25 [0.99, 1.58]	1.34 [0.96, 1.86]	1.08 [0.77, 1.52]	0.309
Child breastfed for at least 6 months	2,677 (34%)	1.24 [0.97, 1.59]	1.22 [0.86, 1.73]	0.94 [0.65, 1.35]	
Parental social class					
Professional, managerial or skilled	6,579 (85%)				
Partly skilled / unskilled	1,178 (15%)	0.95 [0.74, 1.20]	1.16 [0.85, 1.60]	0.98 [0.67, 1.42]	0.736
Maternal educational attainment					
A-level/degree (ref)	3,399 (41%)				
O-level	2,916 (35%)	0.92 [0.76, 1.12]	0.82 [0.62, 1.08]	1.16 [0.87, 1.54]	
Vocational or none	1,909 (23%)	0.82 [0.66, 1.03]	0.94 [0.70, 1.26]	0.92 [0.65, 1.30]	0.358
Material hardship at 33 months[†]					
No material hardship [score<5] (ref.)	5,733 (73%)				
Material hardship [score ≥ 5]	2,084 (27%)	1.00 [0.82, 1.22]	1.06 [0.81, 1.40]	1.52 [1.15, 2.00]	0.026
Home ownership at 33 months					

Confidential: For Review Only

Home owned/mortgaged (ref.)	6,524 (85%)				
Privately rented	312 (4.1%)	0.73 [0.44, 1.20]	1.12 [0.62, 2.02]	1.26 [0.69, 2.29]	0.401
Subsidised rented	832 (11%)	0.74 [0.55, 1.00]	1.01 [0.68, 1.48]	0.92 [0.60, 1.43]	
Car access at 33 months					
Yes (ref.)	7,230 (93%)				
No	581 (7%)	0.82 [0.57, 1.18]	1.53 [1.02, 2.27]	1.49 [0.97, 2.29]	0.018
Gestational age at delivery					
≥ 37weeks (ref)	8,000 (95%)				
< 37 weeks	435 (5%)	0.76 [0.50, 1.16]	1.28 [0.80, 2.05]	1.12 [0.66, 1.93]	0.334
Birth weight					
≥ 2500g (ref)	7,964 (96%)				
< 2500g	372 (4%)	1.29 [0.88, 1.88]	1.32 [0.79, 2.21]	1.05 [0.56, 1.97]	0.488
Developmental level at 18m‡					
Per 1 SD reduction in development	7,931	0.95 [0.87, 1.05]	1.44 [1.28, 1.62]	1.31 [1.13, 1.51]	<0.001
Age at initiation of toilet training:					
Unadjusted model					
Before 6 months	168 (2%)	1.20 [0.67, 2.15]	1.50 [0.69, 3.28]	1.73 [0.78, 3.81]	
Between 6 &15 months	1,056 (14%)	1.22 [0.95, 1.56]	1.09 [0.74, 1.59]	0.81 [0.49, 1.32]	<0.001
Between 15 & 24 months (ref)	3,841 (50%)				
After 24 months	2,650(34%)	0.91 [0.75, 1.12]	1.47 [1.13, 1.91]	1.56 [1.17, 2.07]	

†. Material hardship was assessed using the set of questions: “How difficult at the moment do you find it to afford these items? Food, clothing, heating, rent, items for child”: each on a four-point scale from very difficult through to not difficult. Responses were summed and a binary variable was derived to indicate the top 20% of the sample.

‡. Developmental level was assessed using a questionnaire developed by ALSPAC including items from the Denver Developmental Screening Test[24] and comprising four domains of development (fine motor, gross motor, communication and social skills). We used a total development score derived from the sum of the scores on each domain. Scores on each domain were adjusted for age in weeks and standardized (using a linear regression model and extracting the residuals) and reversed where appropriate so that high values on all scores reflected a lower level of development (increase in the odds of membership to the latent classes per one standard deviation increase in developmental level score).

Confidential: For Review Only

There was strong evidence that boys were at greater odds of being a member of the 'soiling alone' and 'constipation with soiling' classes, however at lower odds of experiencing constipation alone. We also found strong evidence that the presence of hard stools (sometimes or usually) at 2½ years was associated with increased odds of membership to the 'constipation alone' class however there was little evidence of hard-stool consistency being associated with constipation with soiling. There was also a suggestion that hard-stools might be protective against the development of 'soiling without constipation' with a reduction in odds of approximately one third.

We found little evidence of an association between breastfeeding duration and later problems with constipation and/or soiling and we found no association between gestational age or birthweight and constipation and soiling classes. For socioeconomic position the pattern of results was inconsistent. We found no evidence that parental social class, maternal educational attainment and home ownership were associated with constipation and soiling. Material hardship, however, was associated with constipation with soiling and lack of car access was associated with soiling alone. Lower developmental level at 18 months was associated with 'soiling alone' and 'constipation and soiling'. However, there was no association between developmental level 'constipation alone'.

Finally, a later age at initiation of toilet training was associated with 'soiling alone' (47% increase: 13%-91%) and 'constipation with soiling' (56%: 17%-107%). These odds ratios were attenuated following adjustment for the confounders: 'soiling alone' (1·30 [0·97, 1·75]), 'constipation with soiling' (1·27 [0·92, 1·75]). The most influential confounders were developmental delay, major financial difficulties, sex (male) (membership of 'soiling alone' class) and maternal depression ('constipation with soiling' class).

Confidential: For Review Only

DISCUSSION

Main findings

We found that the variability in longitudinal data on childhood constipation and soiling years in a large UK birth cohort could each be adequately explained by four latent classes. On cross-classifying these groupings we identified four clinically-relevant longitudinal phenotypes. Three quarters of children were members of the 'normative' class with a very low probability of constipation or soiling across childhood. Two classes comprised children with constipation alone (13·2% of children) and soiling alone (7·5%) and finally, a class of children who had constipation with soiling (4·8%). Among children with soiling, around 60% had soiling alone and around 40% had soiling with constipation. The prevalence of constipation (18%) observed is higher than the median prevalence (8·9%) reported in a systematic review of 0–18-year-olds.[5] Boys were more likely than girls to experience soiling as reported elsewhere.[5,7]

We found that hard stools in early childhood were associated with a twofold increased odds of 'constipation alone' at school age, and a similar reduced-risk for 'soiling alone'. Children with developmental delay had more 'soiling alone' and 'constipation with soiling', but not 'constipation alone'. We found limited evidence for socioeconomic disadvantage and no evidence that a shorter duration of breastfeeding, shorter gestation, lower birthweight, or the timing of toilet training were risk factors for constipation or soiling at school age.

Strengths and weaknesses

A major strength is the availability of repeated measures of constipation and soiling across childhood in a large, representative cohort. Using this data, we extended our previous work by modelling constipation and soiling in parallel. The resultant latent classes allowed us to estimate,

Confidential: For Review Only

the prevalence of soiling with or without constipation and for constipation alone across childhood and, the differences in early risk factors.

A potential limitation is the use of maternal-report measures of constipation and soiling. Parents were asked to report whether their child had suffered from constipation in the past 12 months and whether they saw a doctor. Previous studies report that only a small proportion of children see a doctor for soiling problems,[8] perhaps because parents are unaware that medical advice and treatment is available. Also parents of children who soil may be unaware that their child is constipated. In addition, the questions relating to soiling did not elicit information about quantity so not distinguishing between leakage, normal bowel movement and staining. Some parents added addenda to the questionnaire noting for instance active diarrhoea and poor wiping and did not categorise their child as soiling, so anecdotal evidence indicates that parents are able to distinguish between true soiling and other occurrences.

Parents in the present study were not asked about the duration of constipation or soiling at each time point, however, the repeated measurements of these conditions suggest that constipation and soiling are persisting problems. Information on frequency of soiling was omitted from the latent class models because frequent soiling was rare. Our aim was to describe the trajectories of constipation and soiling in the community and not to focus solely on children whose bowel problems meet the current established diagnostic criteria. Finally, there was no information available on underlying organic causes of constipation and soiling (e.g. Hirschsprung's disease or anorectal malformations), but the majority of cases are functional. Children with soiling and/or constipation have relatives with these problems,[7,28,29] information we did not have.

Confidential: For Review Only

Comparison of our findings with previous studies

Clinicians believe that most cases of childhood soiling are from chronic constipation, however, we found that among soilers, constipation with soiling (39%) was less common than soiling alone (61%). Other estimates for constipation with soiling[2,28] are from clinic samples. Children with soiling present less often to a clinician, perhaps because they feel ashamed or they believe it is due to laziness.[8] Compared to constipation with soiling, which comes with abdominal pain and infrequent stools, soiling may have no other symptoms.[2] In agreement with earlier studies,[11,12] we found that hard stools in early childhood were strongly associated with later constipation. Early hard stools were, however, not associated with soiling alone or constipation *with* soiling, suggesting that soiling is a primary continence issue and not secondary to constipation.

There is evidence from earlier studies that constipation and soiling are more common among children from lower socioeconomic background.[8,9,10,16] We found little evidence that socioeconomic factors are associated with childhood constipation and soiling. Developmental delay was associated with any soiling but not with constipation alone. Constipation alone may be more strongly related to family history, early experiences of painful defecation and defecation anxiety while soiling is related to delays in reaching social developmental milestones. We found no evidence that length of gestation or birthweight were associated with constipation or soiling at school age consistent with an earlier study.[19] After adjusting for confounders, earlier or later initiation of toilet training had no significant effect on constipation or soiling similar to previous studies[19,20] and in contrast to a large cohort study.[7]

Confidential: For Review Only

Possible implications for clinicians and policymakers

This study finds evidence that experiencing hard stools in early childhood is a risk factor for later problems with constipation at school age. Early identification of children at risk of constipation is important because more than a third of cases become chronic and require secondary care.[30] Early diagnosis and treatment will reduce the risk of constipation persisting to school age resulting in poor quality of life. The burden of constipation is large - 13·2% of primary school age children. Extra health care costs are substantial as 5·9% of the ALSPAC cohort 4-9 years of age, saw a doctor at least once (data not shown). Many would have been referred to secondary care and tertiary paediatric gastroenterology and paediatric surgery services.

Unanswered questions and future research

Further population-based studies are needed and if non-retentive soiling rates are confirmed, parents should be encouraged to seek help for soiling from clinicians who recognise the problem. As with daytime wetting the mainstay of treatment for soiling (alone and the soiling aspect of soiling with constipation) will be behavioural, for example supporting children to attempt to empty their bowels into the toilet at least once per day. This form of therapy requires practitioners to be skilled at providing behavioural interventions concurrently with medications for children who are constipated.

Confidential: For Review Only

Confidential: For Review Only

Acknowledgements and funding:

We are extremely grateful to all the families who took part in this study, the midwives for their help in recruiting them, and the whole ALSPAC team, which includes interviewers, computer and laboratory technicians, clerical workers, research scientists, volunteers, managers, receptionists and nurses. The MRC and Wellcome Trust (Grant ref: 102215/2/13/2) and University of Bristol provide core support for ALSPAC. This publication is the work of the authors who will serve as guarantors for the contents of this paper. This work was supported by the MRC (MR/L007231/1) and NHS Greater Glasgow & Clyde Health Board (*Stephen McLeod* - Head of Specialist Children's Services).

Declaration of interest

The authors have no financial or personal relationships to declare.

Author contributions

Secured funding: DT and CJ

Study design: JH, DT, AvG and CJ

Literature search: CJ

Data analysis: JH and MG

Data interpretation: JH, MG, DT, AvG and CJ

Writing: JH, MG, DT, AvG and CJ

Role of the funding source(s): The funders had no role in the writing of the manuscript or any other aspect pertinent to the study. Authors JH (corresponding author) and MG have full access to all the data.

References

1. Kovacic K, Sood MR, Mugie S, et al. A multicenter study on childhood constipation and fecal incontinence: effects on quality of life. *J Pediatr* 2015; **166**: 1482–7.e1.
2. Bongers ME, Tabbers MM, Benninga MA. Functional nonretentive fecal incontinence in children. *J Pediatr Gastroenterol Nutr* 2007; **44**: 5–13. Review.
3. Hyams JS, Di Lorenzo C, Saps M, Shulman RJ, Staiano A, van Tilburg M. Functional Disorders: Children and Adolescents. *Gastroenterology* 2016: S0016–5085(16)00181–5.
4. Zeevenhooven J, Koppen IJN, Benninga MA. The New Rome IV Criteria for Functional Gastrointestinal Disorders in Infants and Toddlers. *Pediatric Gastroenterology, Hepatology & Nutrition* 2017; **20**: 1–13.
5. van den Berg MM, Benninga MA, Di Lorenzo C. Epidemiology of childhood constipation: a systematic review. *Am J Gastroenterol* 2006; **101**: 2401–9.
6. Mugie SM, Benninga MA, Di Lorenzo C. Epidemiology of constipation in children and adults: a systematic review. *Best Pract Res Clin Gastroenterol* 2011; **25**: 3–18
7. Bellman M. Studies on encopresis. *Acta Paediatr Scand* 1966; **170**: 1– 151
8. van der Wal MF, Benninga MA, Hirasing RA. The prevalence of encopresis in a multicultural population. *J Pediatr Gastroenterol Nutr* 2005; **40**: 345–8.
9. Chung JM, Lee SD, Kang DI, et al; An epidemiologic study of voiding and bowel habits in Korean children: a nationwide multicenter study. *Urology* 2010; **76**: 215–9.
10. Rajindrajith S, Devanarayana NM, Benninga MA. Constipation–associated and nonretentive fecal incontinence in children and adolescents: an epidemiological survey in Sri Lanka. *J Pediatr Gastroenterol Nutr* 2010; **51**: 472–6.

Confidential: For Review Only

11. Loening–Baucke V. Constipation in early childhood: patient characteristics, treatment, and longterm follow up. *Gut* 1993; **34**: 1400–4.

12. Partin JC, Hamill SK, Fischel JE, Partin JS. Painful defecation and fecal soiling in children. *Pediatrics* 1992; **89**: 1007–9.

13. Blum NJ, Taubman B, Osborne ML. Behavioral characteristics of children with stool toileting refusal. *Pediatrics* 1997; **99**: 50–3.

14. Weaver LT, Ewing G, Taylor LC. The bowel habit of milk–fed infants. *J Pediatr Gastroenterol Nutr* 1988; **7**: 568–71.

15. Park M, Bang YG, Cho KY. Risk Factors for Functional Constipation in Young Children Attending Daycare Centers. *J Korean Med Sci* 2016; **31**: 1262–5.

16. Ludvigsson JF1; Abis Study Group. Epidemiological study of constipation and other gastrointestinal symptoms in 8000 children. *Acta Paediatr* 2006; **95**: 573–80.

17. Hack M, Taylor HG, Klein N, Mercuri-Minich N. Functional limitations and special health care needs of 10– to 14–year–old children weighing less than 750 grams at birth. *Pediatrics* 2000; **106**: 554–60.

18. Chaidez V1, Hansen RL, Hertz–Picciotto I. Gastrointestinal problems in children with autism, developmental delays or typical development. *J Autism Dev Disord* 2014; **44**: 1117–27.

19. Largo RH, Molinari L, von Siebenthal K, Wolfensberger U. Development of bladder and bowel control: significance of prematurity, perinatal risk factors, psychomotor development and gender. *Eur J Pediatr* 1999; **158**: 115–22.

20. Blum NJ, Taubman B, Nemeth N. Relationship between age at initiation of toilet training and duration of training: a prospective study. *Pediatrics* 2003; **111**: 810–4.

Confidential: For Review Only

21. Schum TR, Kolb TM, McAuliffe TL, Simms MD, Underhill RL, Lewis M. Sequential acquisition of toilet–training skills: a descriptive study of gender and age differences in normal children. *Pediatrics* 2002; **109**: E48

22. Heron J, Joinson C, Croudace T, von Gontard A. Trajectories of daytime wetting and soiling in a United Kingdom 4 to 9–year–old population birth cohort study. *J Urol* 2008; **179**: 1970–5.

23. Boyd A, Golding J, Macleod J, et al. Cohort Profile: the 'children of the 90s'–the index offspring of the Avon Longitudinal Study of Parents and Children. *Int J Epidemiol* 2013; **42**: 111–27.

24. Frankenburg WK, Dodds J, Archer P, Shapiro H, Bresnick B. The Denver II: a major revision and restandardization of the Denver Developmental Screening Test. *Pediatrics* 1992; **89**: 91–97.

25. Muthén LK, Muthén BO. Mplus User's Guide. 7th Edition: Los Angeles, California: Muthén & Muthén, 2012.

26. Vermunt, J. K. Latent Class Modeling with Covariates: Two Improved Three–Step Approaches. *Political Analysis* 2010; **18**: 450–469.

27. Heron JE, Croudace TJ, Barker ED, Tilling K. A comparison of approaches for assessing covariate effects in latent class analysis. *Longitudinal and Life Course Studies* 2015; **6**: 420–434

28. Benninga MA, Büller HA, Heymans HS, Tytgat GN, Taminiau JA. Is encopresis always the result of constipation? *Arch Dis Child* 1994; **71**: 186–93.

29. Chan AO, Hui WM, Lam KF et al. Familial aggregation in constipated subjects in a tertiary referral center. *Am J Gastroenterol* 2007; **102**: 149–52.

Confidential: For Review Only

30. National Institute for Health and Care Excellence. Constipation in children and young people. https://www.nice.org.uk/guidance/qs62. Date accessed: 19/0917

Confidential: For Review Only

Figure 1. Trajectories for constipation and soiling

Confidential: For Review Only

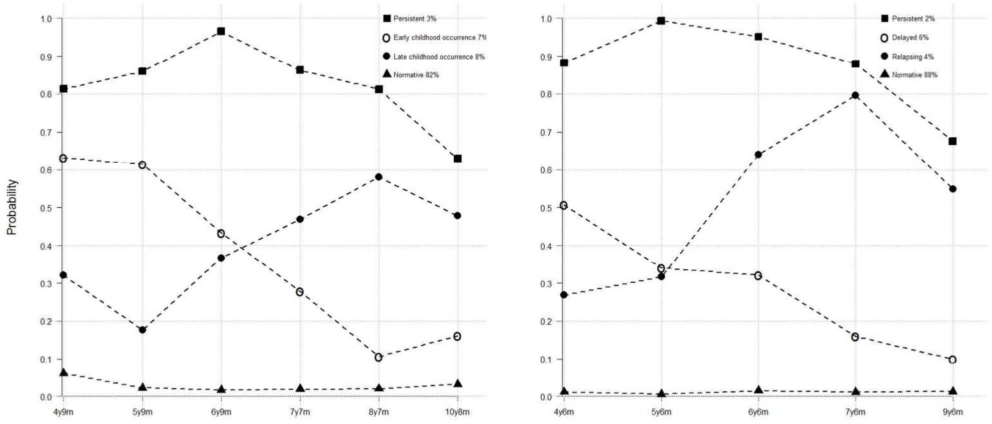

677x339mm (72 x 72 DPI)

For Review Only

Table S1. Goodness of fit statistics for LLCA model of constipation

Sample	# classes	SSA-BIC	Entropy	LMR-p	BLRT-p
Cases with complete data for six measures of constipation (N=5,435)	1 class	21959.6	-	-	-
	2 classes	18334.3	0.878	<0.001	<0.001
	3 classes	18239.7	0.836	<0.001	<0.001
	4 classes	18209.0	0.842	<0.001	<0.001
	5 classes	18229.5	0.863	0.223	0.090
Cases with at least 3 non-missing measures of constipation (N=8,979)	1 class	32429.9	-	-	-
	2 classes	27293.9	0.859	<0.001	<0.001
	3 classes	27159.6	0.808	<0.001	<0.001
	4 classes	27078.8	0.818	<0.001	<0.001
	5 classes	27103.7	0.836	0.661	0.050
Cases with at least 1 non-missing measure of constipation (N=11,195)	1 class	34855.7	-	-	-
	2 classes	29567.9	0.813	<0.001	<0.001
	3 classes	29431.7	0.772	<0.001	<0.001
	4 classes	29342.3	0.785	<0.001	<0.001
	5 classes	29367.8	0.805	0.600	0.050

The LLCA model permits the inclusion of partial respondents (those who provide some but not all repeated measures) through the use of Full Information Maximum Likelihood (FIML) estimation. FIML is a commonly used approach for estimating models within the SEM framework and is often computationally less demanding than Multiple Imputation (MI). Both FIML and MI are based on the Missing At Random assumption. In the case of FIML, MAR equates to the notion that, conditional on the variables in the estimated model, there are no systematic differences between observed and non-observed values of any partially complete variable.

SSA-BIC - sample-size adjusted Bayesian Information Criterion. The Bayesian Information Criterion (BIC) is the traditional fit statistic for comparing mixture models. BIC will typically decrease and then increase following the incremental additional of classes. Using this statistic, the model with the lowest BIC (or other models with BIC values in that vicinity) would be deemed optimal. There is good support for 4-classes using the BIC.

Entropy. This is a measure of classification accuracy, and whilst it is generally of little use in determining the optimal model, it indicates the level of bias which one would expect were a standard three-step estimation to be performed. Here entropy is fairly high and above the commonly-used threshold of 0.8 for all models. We have recently demonstrated (Heron, Croudace, Barker, & Tilling, 2015) that bias can result even for values reaching 0.9 and hence we have opted for the bias-adjusted three-step method as described in the methods.

The Bootstrap Likelihood Ratio Test (BLRT) and the Lo-Mendell-Rubin (LMR) test statistics both assess change in model fit when adding an additional class. Here a high p-value for a k-class model indicates no substantial improvement in fit compared to the k-1 class solution. Whilst the BLRT is usually more conservative, both are in support of a 4-class solution.

Heron, J. E., Croudace, T. J., Barker, E. D., & Tilling, K. (2015). A comparison of approaches for assessing covariate effects in latent class analysis. Longitudinal and Life Course Studies; Vol 6, No 4 (2015): Longitudinal and Life Course Studies.

Confidential: For Review Only

Table S2. Goodness of fit statistics of parallel LLCA models including between 3 and 5 classes both on soiling and constipation dimension.

# soiling classes	# constipation classes	Complete data for both soiling *and* constipation (N=4,931)		At least 3 measures each for both soiling *and* constipation (N=8,435)		At least 1 measure each for both soiling *and* constipation (N=10,450)	
		Entropy	SSA-BIC	Entropy	SSA-BIC	Entropy	SSA-BIC
3	3	0.833	25598.3	0.823	40364.7	0.801	44632.6
3	4	0.843	25574.4	0.832	40298.0	0.810	44563.6
3	5	0.847	25602.4	0.846	40328.6	0.826	44596.9
4	3	0.859	25594.6	0.846	40360.2	0.825	44628.7
4	4	0.879	**25571.9**	0.852	**40285.3**	0.830	**44552.1**
4	5	0.882	25603.4	0.866	40317.3	0.842	44586.0
5	3	0.841	25620.5	0.834	40393.9	0.794	44664.7
5	4	0.884	25597.3	0.870	40321.8	0.850	44589.9
5	5	0.893	25631.1	0.884	40357.0	0.861	44627.6

There was good support for a 4-class solution for both constipation and soiling when considered separately. When both sets of data were brought together for the estimation of a joint model we considered 3-, 4- and 5-class solutions for both dimensions. The reason being that the additional information can distort the estimation of the two latent class measurement models, particularly when missing data is present. In the current situation, there remained good support for a 4-class by 4-class solution and the individual trajectories estimated for soiling and constipation were not adversely affected.

Confidential: For Review Only

Table S3. Association between risk factors and the latent classes of (i) Constipation, and (ii) Soiling

	(i) Constipation classes				(ii) Soiling classes			
	Early Childhood Occurrence OR [95% CI]	Late Childhood Occurrence OR [95% CI]	Persistent OR [95% CI]	Omnibus p-value	Delayed OR [95% CI]	Relapse OR [95% CI]	Persistent OR [95% CI]	Omnibus p-value
Hard stools regularity at 2.5 yrs				<0.001				0.146
Never hard (ref.)	1.00 ref				1.00 ref			
Sometimes	2.28 [1.32, 3.92]	1.47 [0.99, 2.20]	1.88 [1.11, 3.18]		1.00 [0.55, 1.81]	1.27 [0.75, 2.15]	0.79 [0.52, 1.19]	
Usually hard	2.24 [1.26, 3.98]	1.30 [0.83, 2.03]	2.19 [1.27, 3.78]		0.84 [0.43, 1.64]	0.74 [0.39, 1.40]	0.73 [0.46, 1.17]	
Breastfeeding by 6 months				0.121				0.588
child never breastfed (ref.)	1.00 ref				1.00 ref			
child breastfed for < 6mn	1.03 [0.69, 1.54]	1.46 [0.98, 2.16]	1.05 [0.71, 1.55]		1.30 [0.73, 2.32]	1.52 [0.87, 2.68]	0.85 [0.56, 1.28]	
child breastfed for at least 6mn	1.35 [0.90, 2.00]	1.39 [0.92, 2.10]	0.75 [0.48, 1.19]		1.12 [0.60, 2.09]	1.30 [0.71, 2.38]	0.99 [0.65, 1.50]	
Sex				<.001				<.001
Female (ref.)	1.00 ref				1.00 ref			
Male	0.81 [0.61 1.07]	0.64 [0.49, 0.84]	0.84 [0.62, 1.13]		1.45 [0.95, 2.22]	1.91 [1.30, 2.80]	2.00 [1.42, 2.83]	
Parental social class				0.066				0.721
Non-manual (ref)	1.00 ref				1.00 ref			
Manual	0.81 [0.52, 1.25]	0.68 [0.44, 1.05]	1.43 [0.98, 2.07]		0.76 [0.38, 1.55]	1.19 [0.74, 1.91]	1.16 [0.74, 1.80]	
Maternal educational attainment				0.121				0.795
A-level/degree (ref.)	1.00 ref				1.00 ref			
O-level	0.84 [0.60, 1.17]	0.88 [0.65, 1.18]	1.32 [0.93, 1.89]		1.07 [0.67, 1.70]	0.84 [0.55, 1.30]	0.91 [0.62, 1.32]	
< O-level	0.82 [0.57, 1.19]	0.64 [0.44, 0.94]	1.22 [0.82, 1.82]		0.67 [0.35, 1.28]	1.07 [0.69, 1.66]	1.00 [0.66, 1.51]	
Material hardship at 33 months				0.144				0.087
no hardship [score<5] (ref.)	1.00 ref				1.00 ref			
material hardship [score ≥ 5]	1.18 [0.86, 1.63]	1.00 [0.73, 1.37]	1.38 [0.99, 1.92]		0.83 [0.48, 1.42]	1.11 [0.74, 1.68]	1.53 [1.07, 2.18]	

Confidential: For Review Only

Table S3. Continued

	(i) Constipation class				(ii) Soiling class			
	Early Childhood Occurrence OR [95% CI]	Late Childhood Occurrence OR [95% CI]	Persistent OR [95% CI]	Omnibus p-value	Delayed OR [95% CI]	Relapse OR [95% CI]	Persistent OR [95% CI]	Omnibus p-value
Home ownership at 33 months				0.022				0.791
Home owned/mortgaged (ref.)	1.00 ref				1.00 ref			
Privately rented	0.89 [0.42, 1.86]	0.58 [0.24, 1.41]	0.97 [0.42, 2.21]		1.05 [0.35, 3.16]	1.27 [0.56, 2.88]	0.89 [0.34, 2.35]	
Subsidised rented	0.77 [0.46, 1.28]	0.47 [0.25, 0.90]	1.73 [1.17, 2.57]		0.88 [0.43, 1.80]	0.56 [0.24, 1.28]	1.14 [0.69,1.88]	
Car ownership at 33 months				0.510				0.057
yes, car owned (ref.)	1.00 ref				1.00 ref			
no car	1.28 [0.79, 2.07]	0.65 [0.33, 1.26]	1.16 [0.67, 2.01]		1.32 [0.63, 2.77]	0.89 [0.40, 1.97]	1.90 [1.16, 3.09]	
Gestation age at delivery				0.248				0.309
≥ 37weeks (ref.)	1.00 ref				1.00 ref			
< 37 weeks	0.97 [0.84, 1.11]	0.49 [0.21, 1.14]	0.79 [0.39, 1.60]		1.49 [0.67, 3.27]	1.12 [0.50, 2.51]	1.50 [0.81, 2.77]	
Birth weight				0.075				0.467
≥ 2500g (ref.)	1.00 ref				1.00 ref			
< 2500g	1.13 [0.84, 1.53]	0.84 [0.72, 0.99]	1.38 [0.80, 2.37]		0.73 [0.21, 2.60]	1.23 [0.56, 2.68]	1.56 [0.83, 2.94]	
Developmental level at 18m				0.189	1.00 ref			<0.001
Per 1 SD reduction in development	0.97 [0.81, 1.18]	0.91 [0.77, 1.07]	1.21 [1.01, 1.46]		1.27 [1.04, 1.54]	1.31 [1.05, 1.63]	1.66 [1.39, 2.00]	
Toilet training initiation				0.991				0.012
Before 6mn	1.31 [0.52, 3.29]	1.16 [0.47, 2.85]	1.21 [0.44, 3.35]		1.91 [0.54, 6.78]	2.01 [0.78, 5.15]	1.13 [0.30, 4.30]	
Between 6 &15mn	1.01 [0.63, 1.59]	1.10 [0.74, 1.64]	1.00 [0.61, 1.62]		1.26 [0.66, 2.42]	0.70 [0.35, 1.41]	0.99 [0.56, 1.74]	
Started between 15 & 24mn (ref.)	1.00 ref				1.00 ref			
Started after 24mn	1.13 [0.82, 1.55]	0.90 [0.66, 1.24]	0.99 [0.70, 1.41]		1.60 [0.99, 2.58]	1.15 [0.76, 1.74]	1.62 [1.12, 2.33]	

Confidential: For Review Only

Figure S1. Rates of daytime wetting, bedwetting, stomach ache and infrequent bowel movements within classes of children defined by their constipation and daytime soiling in mid-childhood.

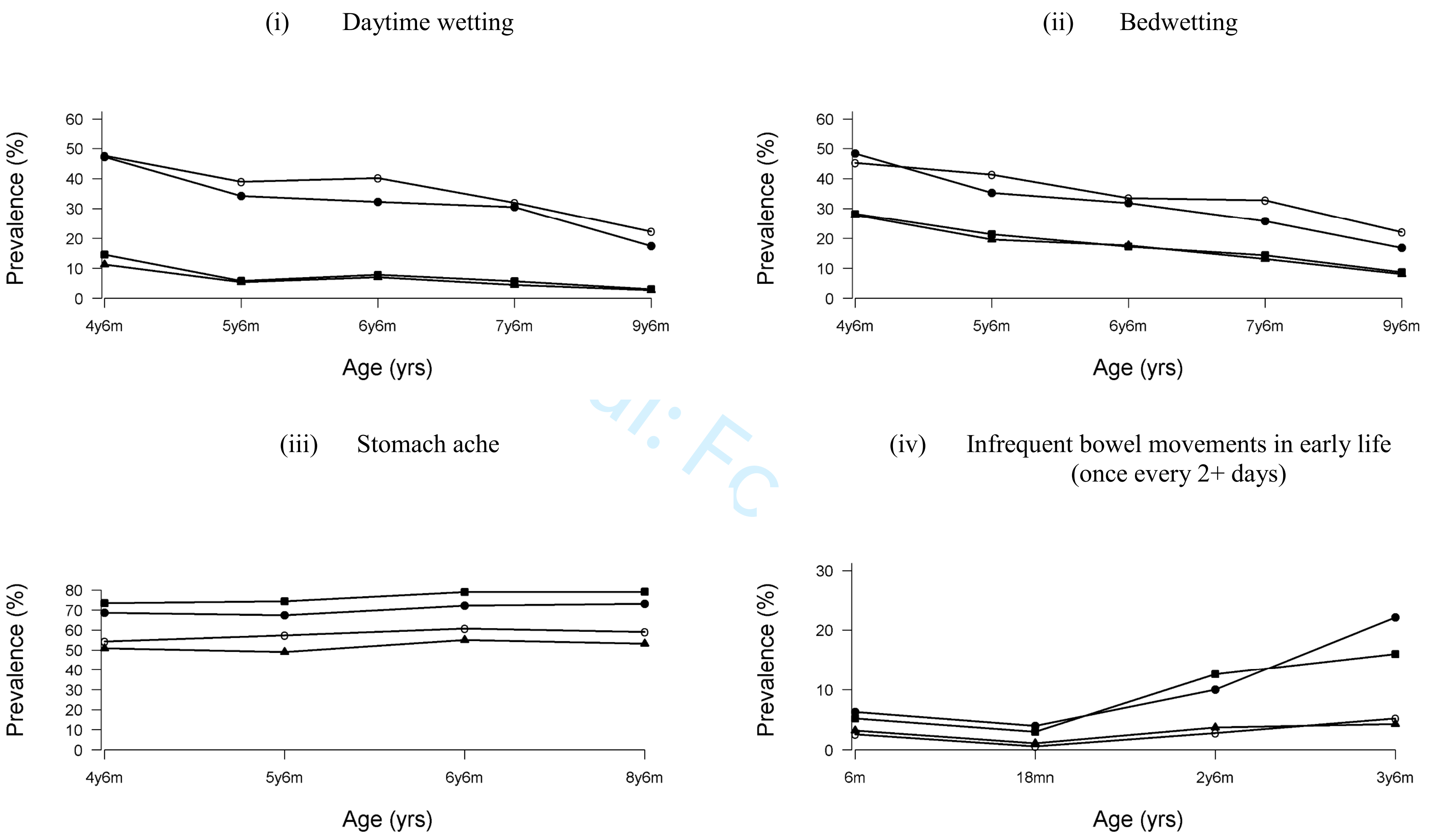

Constipation with soiling (●), Soiling alone (O), Constipation alone (■), Normative (▲)

BMJ Paediatrics Open

Confidential: For Review Only

Early childhood risk factors for constipation and soiling at school age: an observational cohort study

Journal:	*BMJ Paediatrics Open*
Manuscript ID	bmjpo-2017-000230.R1
Article Type:	Original article
Date Submitted by the Author:	04-Jan-2018
Complete List of Authors:	Heron, Jon; University of Bristol, Population Health Sciences<br>Grzeda, Mariusz; University of Bristol, Population Health Sciences<br>Tappin, David; University of Glasgow, Scottish Cot Death Trust, Child Health, School of Medicine<br>von Gontard, Alexander; Saarland University Hospital, Department of Child and Adolescent Psychiatry<br>Joinson, Carol; University of Bristol, Population Health Sciences
Keywords:	Gastroenterology

SCHOLARONE™
Manuscripts

Early childhood risk factors for constipation and soiling at school age: an observational cohort study

Jon Heron[1], Mariusz Grzeda[1], David Tappin[2], Alexander von Gontard[3], Carol Joinson[1]

[1] Department of Population Health Sciences, Bristol Medical School, University of Bristol, UK.

[2] Scottish Cot Death Trust, Child Health, School of Medicine, University of Glasgow, UK.

[3] Department of Child and Adolescent Psychiatry, Saarland University Hospital, Homburg, Germany.

Corresponding author

Dr Jon Heron

Population Health Sciences, Bristol Medical School, University of Bristol

Oakfield House, Oakfield Grove, Bristol, BS8 2BN

Tel: 0117 3310093

Email: jon.heron@bristol.ac.uk

Keywords: ALSPAC.

It is a requirement of the data providers that we include ALSPAC as a keyword however this was not permitted by the online submission system.

Confidential: For Review Only

A. What is known about the subject

Constipation and soiling are common in childhood, but little is known about factors that increase the risk of these problems persisting at primary school age.

The majority of earlier studies examining risk factors for childhood constipation and soiling are cross-sectional and many are based on small / clinic samples.

No prospective cohort studies have examined the association between risk factors in early childhood and different trajectories of constipation and soiling at primary school age.

B. What this study adds

This study finds evidence that risk factors in early childhood are differentially associated with different trajectories of childhood constipation and soiling.

Contrary to common beliefs of clinicians, we found that among children with soiling, those with soiling alone (i.e. without constipation) outnumbered those with associated constipation.

Experiencing hard stools in early childhood is a risk factor for constipation; developmental delay is a risk factor for soiling alone and constipation with soiling.

Confidential: For Review Only

ABSTRACT

Objective. Constipation and soiling are common in childhood. This study examines the co-morbidity between childhood constipation and soiling and early childhood risk factors for these problems.

Design. The sample comprised 8,435 participants from the Avon Longitudinal Study of Parents and Children with maternally-reported measures of constipation (6 time-points between 4–10yrs) and soiling (5 time-points between 4–9yrs). We used latent class analysis to extract longitudinal patterns of constipation and soiling. We examined whether the latent classes are differentially associated with maternally reported risk factors in early childhood (stool consistency, breastfeeding, socioeconomic background, gestation, birthweight, developmental level and age at initiation of toilet training) using multinomial logistic regression models.

Results. We extracted 4 latent classes: 'normative' (74·5%: very low probability of constipation or soiling); 'constipation alone' (13·2%); 'soiling alone' (7·5%) and 'constipation with soiling' (4·8%). Hard stools at 2½ years were associated with increased odds of constipation alone. Developmental delay at 18 months was associated soiling alone and constipation with soiling, but not constipation alone. We found limited evidence of associations with socioeconomic background and no evidence of associations with age at initiation of toilet training, breastfeeding, gestational age or birthweight.

Conclusion. Constipation alone was the most prevalent pattern in this cohort. Treatment for hard stools in early childhood is needed to prevent chronic constipation at school age. Constipation with soiling was less common than soiling alone. Further research is needed into the causes of non-retentive soiling.

Confidential: For Review Only

Confidential: For Review Only

INTRODUCTION

Bowel problems are common in childhood and have a considerable impact on quality of life.[1] It is believed that 80% of faecal incontinence is due to overflow from chronic constipation, while 20% have no constipation (functional non-retentive fecal incontinence).[2] The Rome-IV definition for functional constipation at developmental age 4 years and above requires at least two of six symptoms (2 or fewer defecations in the toilet per week; at least 1 episode of fecal incontinence per week; history of retentive posturing/stool retention; history of painful or hard stools; a large fecal mass in the rectum; large diameter stools that can obstruct the toilet) present once a week or more for at least one month.[3] Rome-IV diagnostic criteria are also available for functional constipation in children under 4 years.[4] The diagnostic criteria for functional non-retentive faecal incontinence are: inappropriate defecation; no medical condition for symptoms, and no retention (criteria should be met for at least one month). [3] Other clinical definitions are sometimes used.[5,6] Epidemiological studies of the prevalence of constipation and soiling vary probably because of different definitions. A systematic review reported the median prevalence of constipation in 0–18-year-olds to be 8·9%, with similar prevalence in boys and girls,[5] however, more recent findings suggest a higher proportion of constipation in girls.[6] The prevalence of childhood soiling is between 1% and 4% and is consistently found to be two to four times more common in boys.[7,8] A recent large cross-sectional study of 5-13-year-olds reported that 7·8% (9·8% boys, 5·8% girls) experienced faecal incontinence.[9] Only one epidemiological survey, of 10–16-year-olds in Sri Lanka, differentiated between soiling with and without constipation and reported that 2·0% experienced faecal incontinence and 18% of those did not have constipation.[10]

Early identification of children at risk of constipation and soiling could lead to timely interventions to reduce the adverse impacts on quality of life and psychosocial development. Clinicians believe that pain of passing hard stools in infancy and early childhood is the principal contributing factor for acute childhood constipation[11] leading to chronic constipation which causes soiling.[11,12] Hard stools lead to withholding and toileting refusal,[13] retaining a stool mass and increasing the difficulty of evacuating. Breastfed infants produce softer stools[14] and those breastfed for less than 6 months may develop constipation more commonly.[15] Other risk factors include lower levels of parental education,[9,16] income[9] and socioeconomic status,[8,10] low birthweight and prematurity[17] and developmental delay.[18] Timing of toilet training has also been investigated but findings are inconsistent.[7,19,20] Only one study specified whether constipation occurred with or without soiling.[11] Very little is known about risk factors for soiling without constipation. Finally, most earlier studies of risk factors for constipation and soiling are cross-sectional which makes the timing of events more difficult to determine.

Although most children achieve bowel control by 3–5 years,[19,21] there is recent evidence for different patterns of development of bowel control.[22] These ‘developmental trajectories’ distinguish children with normative development (89·0%), delayed attainment (4·1%), persistent soiling (2·7%) and relapses in soiling (4·1%).[22] Describing developmental trajectories of soiling alone does not allow the determination of whether soiling is occurring with or without constipation. The aims of this paper are twofold: First, we extend previous work using data from the ALSPAC birth cohort to determine the degree of co-morbidity between constipation and soiling in childhood and second, we examine the association between risk factors in early childhood and trajectories of constipation and soiling at primary school age.

Confidential: For Review Only

Confidential: For Review Only

METHODS

Participants

The sample comprised participants from the Avon Longitudinal Study of Parents and Children (ALSPAC). Detailed information is available at (http://www.bristol.ac.uk/alspac), including a fully searchable data-dictionary (http://www.bris.ac.uk/alspac/researchers/data-access/data-dictionary). Pregnant women resident in the former Avon Health Authority in SW England, having an estimated date of delivery between 1/4/91 and 31/12/92 were invited to take part, resulting in a cohort of 14,541 pregnancies.[23] Of the 13,978 singletons/twins alive at one year, 24 participants withdrew consent leaving a starting sample of 13,954. Ethical approval was obtained from the ALSPAC Law and Ethics committee and local research ethics committees.

Soiling and constipation in mid-childhood

When children were 4½, 5½, 6½, 7½ and 9½ years their parents were asked: "How often usually does your child dirty his/her pants during the day?" with options: 'Never'; 'Occasional accident but less than once/week'; 'About once/week'; '2-5 times/week'; 'Nearly every day'; and 'More than once a day'. Responses were collapsed ('Never' versus all other responses) to indicate presence of soiling at each age. Parents were also asked about their child's constipation across a similar age range: "Has he/she had any constipation in the past 12 months?" with options: 'Yes, and saw a doctor'; 'Yes, but did not see the doctor', and 'No, did not have'. Responses were collapsed (Yes with/without consultation versus No) to indicate presence of constipation at each age.

Risk factors in early childhood

Potential risk factors were identified from the literature. Data were obtained from questionnaires completed by mothers and included the child's stool consistency at 2½ years, breastfeeding

during the first six months, family socioeconomic position assessed during the antenatal period (parental social class, maternal educational attainment) or at 33 months (material hardship, home ownership and car access), length of gestation and birthweight, developmental level[24] and age at initiation of toilet training (assessed when the child was 6, 15, and 24 months).

Statistical modelling

We have previously used longitudinal Latent Class Analysis (LLCA) to derive developmental trajectories of soiling[22] where we showed that patterns of soiling at 4–9 years could be adequately explained by a four-class solution. However, such a model ignores the comorbidity with constipation. Therefore, in the current study we (i) estimated a similar latent class model of for constipation and (ii) combined this with soiling in a parallel model to describe changes in both problems (using Mplus v7·11[25]). Further details regarding the estimation can be found in the Supplementary Appendix. In addition, as various sample sizes have been used for the different analytical steps. Supplementary Figure S1 shows a flowchart describing how each sample was obtained.

Once the optimal model had been established, we examined the association between the risks (above) and constipation/soiling class-membership using multinomial logistic regression. Coefficients and standard errors were estimated using the bias-adjusted three-step approach[26,27] which has been shown to reduce the bias inherent in such regression models.[26] For most risks we report univariable estimates of association, however for toilet training initiation we considered the potential confounding effects of gender, social class, early parenthood, mother's education, housing adequacy, major financial difficulties, family size, social network/support, developmental delay, and maternal depression.

Confidential: For Review Only

RESULTS

Table 1 shows rates of soiling and constipation at each age. Whilst rates of reported constipation decrease steadily, there is a more complex pattern for soiling. Of those reporting constipation, the proportion who saw a doctor decreased from 19·7% to 11·6% (data not shown).

Confidential: For Review Only

Table 1. Prevalence of soiling and constipation in the ALSPAC study

Subset of dataset containing at least one non-missing time points for both soiling and constipation (max N = 10,450)

	4½ years	5½ years	6½ years	7½ years	9½ years	
No soiling	8720 (92.8%)	8284 (93.8%)	7752 (92.2%)	7617 (93.1%)	7288 (94.8%)	
Soiling	673 (7.2%)	548 (6.2%)	654 (7.8%)	565 (6.9%)	397 (5.2%)	
	4years 9mn	5years 9mn	6years 9mn	7years 7mn	8years 7mn	10years 8mn
No constipation	7947 (85.6%)	7622 (89.6%)	7580 (89.7%)	7346 (89.9%)	7062 (90.2%)	6631 (90.4%)
Constipation	1342 (14.4%)	881 (10.4%)	873 (10.3%)	822 (10.1%)	765 (9.8%)	707 (9.6%)

Subset of dataset containing at least three non-missing time points for both soiling and constipation, (max N=8,435)

	4½ years	5½ years	6½ years	7½ years	9½ years	
No soiling	7,507 (92.8%)	7,518 (94.0%)	7,268 (92.2%)	7,153 (93.2%)	6,801 (94.8%)	
Soiling	585 (7.2%)	477 (6.0%)	611 (7.8%)	525 (6.8%)	372 (5.2%)	
	4years 9mn	5years 9mn	6years 9mn	7years 7mn	8years 7mn	10years 8mn
No constipation	6,893 (85.5%)	7,014 (89.7%)	7,086 (89.6%)	6,894(90.0%)	6,549 (90.5%)	6,194 (90.6%)
Constipation	1,173 (14.5%)	809 (10.3%)	825 (10.4%)	766 (10.0%)	689 (9.5%)	644 (9.4%)

Sample with complete data on all eleven measures, (N=4,931)

	4½ years	5½ years	6½ years	7½ years	9½ years	
No soiling	4575 (92.8%)	4638 (94.1%)	4568 (92.6%)	4590 (93.1%)	4670 (94.7%)	
Soiling	356 (7.2%)	293 (5.9%)	363 (7.4%)	341 (6.9%)	261 (5.3%)	
	4years 9mn	5years 9mn	6years 9mn	7years 7mn	8years 7mn	10years 8mn
No constipation	4244 (86.1%)	4425 (89.7%)	4402 (89.3%)	4426 (89.8%)	4454 (90.3%)	4458 (90.4%)
Constipation	687 (13.9%)	506 (10.3%)	529 (10.7%)	505 (10.2%)	477 (9.7%)	473 (9.6%)

Confidential: For Review Only

Unconditional model for constipation

There was good support for a 4-class solution when considering the six repeated measurements of constipation. Focussing on the sample consisting of cases with at least 3 non-missing measures of constipation (n=8,979) the following classification of children was obtained: Normative (82%; very low probability of constipation throughout), Early childhood occurrence (7%; children suffering from constipation until 6 years), Late childhood occurrence (8% problem with constipating emerging after 6 years), Persistent (3%; high probability of problems throughout).

Parallel model for soiling and constipation

The next step was to merge the two LLCA models into a single parallel model permitting an investigation into the longitudinal association between soiling and constipation- see **Table 2** and **Figure 1**. Based on these results we collapsed the 16 groups defined by such a two-way classification into four clinical relevant subgroups to facilitate further study. The four resulting groups were: *Normative* (74·5% of total) - Normative classes for both constipation and soiling; *constipation alone* (13·2%) - Normative class for soiling and non-normative classes for constipation; *soiling alone* (7·5%) - Normative class for constipation and non-normative classes for soiling; *constipation with soiling* (4·8%) – classified as non-normative in terms of the progression of both constipation and soiling during the period of childhood studied.

Confidential: For Review Only

Table 2. Associations between constipation and soiling: Joint distribution of classes derived from parallel LLCA model (n=8,435).

	Soiling classes				
**Constipation classes**	Normative (soiling)	Delayed	Relapse	Persistent	Total
Normative (constipation)	74.5%[a]	4.3%[c]	2.2%[c]	1.0%[c]	82%
Early childhood occurrence	5.5%[b]	1.1%[d]	<0.1%[d]	0.5%[d]	7.1%
Late childhood occurrence	5.8%[b]	0.6%[d]	0.9%[d]	0.3%[d]	7.6%
Persistent	1.9%[b]	0.4%[d]	0.5%[d]	0.5%[d]	3.3%
Total	87.7%	6.4%	3.6%	2.3%	100%

Footnote:

Data shown in the table are class proportions for the latent class patterns based on the estimated model. These results are for the sample of children with both constipation and soiling data for at least three time points.

[a] Normative (74.5% of total) - Normative classes for both constipation and soiling

[b] Constipation alone (13.2% of total) - Normative class for soiling and non-normative classes for constipation

[c] Soiling alone (7.5% of total) - Normative class for constipation and non-normative classes for soiling

[d] Constipation with soiling (4.8% of total) - Non-normative classes for constipation and soiling

Confidential: For Review Only

Rates of associated symptoms within each class

To gain further insight into this classification we examined the extent to which the rates of daytime wetting, bedwetting and stomach ache in mid-childhood, and infrequent bowel movements in early-childhood differed across the four composite classes (Supplementary Figure S2). Firstly, the two classes involving soiling exhibited similar rates of both daytime wetting and bedwetting and furthermore these rates were consistently higher than the constipation only and the normative class. Secondly, rates of stomach ache were lower for the soiling alone class compared to either class reporting constipation. Finally, in terms of the rates of infrequent bowel movements in early-childhood, those classified as soiling alone were no different to the normative class for whom infrequent movements were rare, whereas both classes experiencing constipation were seen to have infrequent bowel movements which became a more common finding as the children became older.

Risk factors for Constipation / Soiling class membership

Table 3 shows the association between the risk factors and odds-ratios for membership of the classes of 'constipation alone', 'soiling alone' and 'constipation with soiling' with reference to the normative class. For completeness, we append the results for the separate models of soiling and constipation (Supplementary Table S3).

Confidential: For Review Only

Table 3. Association between risk factors and the parallel classes of soiling and constipation (n ≤ 8,435 depending on risk factor)

	n (%)	Constipation alone OR [95% CI]	Soiling alone OR [95% CI]	Constipation with soiling OR [95% CI]	Omnibus p-value
Sex					
Female (ref)	4,082 (52%)				
Male	4,353 (48%)	0.63 [0.53, 0.75]	1.78 [1.39, 2.27]	1.38 [1.06, 1.79]	<0.001
Hard stools regularity at 2½ years					
Never hard (ref)	1,414 (18%)				
Sometimes	4,312 (54%)	2.08 [1.56, 2.77]	1.12 [0.82, 1.52]	1.22 [0.84, 1.78]	<0.001
Usually hard	2,235 (28%)	1.90 [1.40, 2.60]	0.68 [0.46, 0.99]	1.26 [0.85, 1.89]	
Breastfeeding					
Child never breastfed (ref)	1,695 (21%)				
Child breastfed for < 6 months	3,579 (45%)	1.25 [0.99, 1.58]	1.34 [0.96, 1.86]	1.08 [0.77, 1.52]	0.309
Child breastfed for at least 6 months	2,677 (34%)	1.24 [0.97, 1.59]	1.22 [0.86, 1.73]	0.94 [0.65, 1.35]	
Parental social class					
Professional, managerial or skilled	6,579 (85%)				
Partly skilled / unskilled	1,178 (15%)	0.95 [0.74, 1.20]	1.16 [0.85, 1.60]	0.98 [0.67, 1.42]	0.736
Maternal educational attainment					
A-level/degree (ref)	3,399 (41%)				
O-level	2,916 (35%)	0.92 [0.76, 1.12]	0.82 [0.62, 1.08]	1.16 [0.87, 1.54]	
Vocational or none	1,909 (23%)	0.82 [0.66, 1.03]	0.94 [0.70, 1.26]	0.92 [0.65, 1.30]	0.358
Material hardship at 33 months†					
No material hardship [score<5] (ref.)	5,733 (73%)				
Material hardship [score ≥ 5]	2,084 (27%)	1.00 [0.82, 1.22]	1.06 [0.81, 1.40]	1.52 [1.15, 2.00]	0.026
Home ownership at 33 months					

Confidential: For Review Only

Home owned/mortgaged (ref.)	6,524 (85%)				
Privately rented	312 (4.1%)	0.73 [0.44, 1.20]	1.12 [0.62, 2.02]	1.26 [0.69, 2.29]	0.401
Subsidised rented	832 (11%)	0.74 [0.55, 1.00]	1.01 [0.68, 1.48]	0.92 [0.60, 1.43]	
Car access at 33 months					
Yes (ref.)	7,230 (93%)				
No	581 (7%)	0.82 [0.57, 1.18]	1.53 [1.02, 2.27]	1.49 [0.97, 2.29]	0.018
Gestational age at delivery					
≥ 37weeks (ref)	8,000 (95%)				
< 37 weeks	435 (5%)	0.76 [0.50, 1.16]	1.28 [0.80, 2.05]	1.12 [0.66, 1.93]	0.334
Birth weight					
≥ 2500g (ref)	7,964 (96%)				
< 2500g	372 (4%)	1.29 [0.88, 1.88]	1.32 [0.79, 2.21]	1.05 [0.56, 1.97]	0.488
Developmental level at 18m‡					
Per 1 SD reduction in development	7,931	0.95 [0.87, 1.05]	1.44 [1.28, 1.62]	1.31 [1.13, 1.51]	<0.001
Age at initiation of toilet training:					
Unadjusted model					
Before 6 months	168 (2%)	1.20 [0.67, 2.15]	1.50 [0.69, 3.28]	1.73 [0.78, 3.81]	
Between 6 &15 months	1,056 (14%)	1.22 [0.95, 1.56]	1.09 [0.74, 1.59]	0.81 [0.49, 1.32]	<0.001
Between 15 & 24 months (ref)	3,841 (50%)				
After 24 months	2,650(34%)	0.91 [0.75, 1.12]	1.47 [1.13, 1.91]	1.56 [1.17, 2.07]	

†. Material hardship was assessed using the set of questions: "How difficult at the moment do you find it to afford these items? Food, clothing, heating, rent, items for child": each on a four-point scale from very difficult through to not difficult. Responses were summed and a binary variable was derived to indicate the top 20% of the sample.

‡. Developmental level was assessed using a questionnaire developed by ALSPAC including items from the Denver Developmental Screening Test[24] and comprising four domains of development (fine motor, gross motor, communication and social skills). We used a total development score derived from the sum of the scores on each domain. Scores on each domain were adjusted for age in weeks and standardized (using a linear regression model and extracting the residuals) and reversed where appropriate so that high values on all scores reflected a lower level of development (increase in the odds of membership to the latent classes per one standard deviation increase in developmental level score).

Confidential: For Review Only

There was strong evidence that boys were at greater odds of being a member of the 'soiling alone' and 'constipation with soiling' classes, however at lower odds of experiencing constipation alone. We also found strong evidence that the presence of hard stools (sometimes or usually) at 2½ years was associated with increased odds of membership to the 'constipation alone' class however there was little evidence of hard-stool consistency being associated with constipation with soiling. There was also a suggestion that hard-stools might be protective against the development of 'soiling without constipation'.

We found little evidence of an association between breastfeeding duration and later problems with constipation and/or soiling and we found no association between gestational age or birthweight and constipation and soiling classes. For socioeconomic position the pattern of results was inconsistent. We found no evidence that parental social class, maternal educational attainment and home ownership were associated with constipation and soiling. Material hardship, however, was associated with constipation with soiling and lack of car access was associated with soiling alone. Lower developmental level at 18 months was associated with 'soiling alone' and 'constipation and soiling'. However, there was no association between developmental level 'constipation alone'.

Finally, a later age at initiation of toilet training was associated with 'soiling alone' and 'constipation with soiling'. Estimates were attenuated following adjustment for the confounders: 'soiling alone' (adjusted OR = 1.30 [0.97, 1.75]), 'constipation with soiling' (adjusted OR = 1.27 [0.92, 1.75]). The most influential confounders were developmental delay, major financial difficulties, sex (male) (membership of 'soiling alone' class) and maternal depression ('constipation with soiling' class).

Confidential: For Review Only

DISCUSSION

We found that the variability in longitudinal data on childhood constipation and soiling years in a large UK birth cohort could each be adequately explained by four latent classes. On cross-classifying these groupings we identified four clinically-relevant longitudinal classes. Three quarters of children were members of the ‘normative’ class with a very low probability of constipation or soiling across childhood. Two classes comprised children with constipation alone (13·2% of children) and soiling alone (7·5%) and finally, a class of children who had constipation with soiling (4·8%). Among children with soiling, around 60% had soiling alone and around 40% had soiling with constipation. The prevalence of constipation (18%) observed is higher than the median prevalence (8·9%) reported in a systematic review of 0–18-year-olds.[5] Boys were more likely than girls to experience soiling as reported elsewhere.[5,7]

We found that hard stools in early childhood were associated with an increased odds of ‘constipation alone’ at school age, as well as a reduced-risk for ‘soiling alone’. Children with developmental delay had more ‘soiling alone’ and ‘constipation with soiling’, but not ‘constipation alone’. We found limited evidence for socioeconomic disadvantage and no evidence that a shorter duration of breastfeeding, shorter gestation, lower birthweight, or the timing of toilet training were risk factors for constipation or soiling at school age.

A major strength is the availability of repeated measures of constipation and soiling across childhood in a large, representative cohort. Using this data, we extended our previous work by modelling constipation and soiling in parallel. The resultant latent classes allowed us to estimate, the prevalence of soiling with or without constipation and for constipation alone across childhood and, the differences in early risk factors.

Confidential: For Review Only

A potential limitation is the use of maternal-report measures of constipation and soiling. Parents were asked to report whether their child had suffered from constipation in the past 12 months and whether they saw a doctor. Previous studies report that only a small proportion of children see a doctor for soiling problems,[8] perhaps because parents are unaware that medical advice and treatment is available. Also, parents of children who soil may be unaware that their child is constipated. In addition, the questions relating to soiling did not elicit information about quantity so not distinguishing between leakage, normal bowel movement and staining. Some parents added addenda to the questionnaire noting for instance active diarrhoea and poor wiping and did not categorise their child as soiling, so anecdotal evidence indicates that parents are able to distinguish between true soiling and other occurrences.

Parents in the present study were not asked about the duration of constipation or soiling at each time point, however, the repeated measurements of these conditions suggest that constipation and soiling are persisting problems. Information on frequency of soiling was omitted from the latent class models because frequent soiling was rare. Our aim was to describe the trajectories of constipation and soiling in the community and not to focus solely on children whose bowel problems meet the current established diagnostic criteria. Finally, there was no information available on underlying organic causes of constipation and soiling (e.g. Hirschsprung's disease or anorectal malformations), but the majority of cases are functional. Children with soiling and/or constipation have relatives with these problems,[7,28,29] information we did not have.

Clinicians believe that most cases of childhood soiling are from chronic constipation, however, we found that among soilers, constipation with soiling (39%) was less common than soiling alone (61%). Other estimates for constipation with soiling[2,28] are from clinic samples. Children with soiling present less often to a clinician, perhaps because they feel ashamed or they

Confidential: For Review Only

believe it is due to laziness.[8] Compared to constipation with soiling, which comes with abdominal pain and infrequent stools, soiling may have no other symptoms.[2] In agreement with earlier studies,[11,12] we found that hard stools in early childhood were strongly associated with later constipation. Early hard stools were, however, not associated with soiling alone or constipation *with* soiling, suggesting that soiling is a primary continence issue and not secondary to constipation.

There is evidence from earlier studies that constipation and soiling are more common among children from lower socioeconomic background.[8,9,10,16] We found little evidence that socioeconomic factors are associated with childhood constipation and soiling. Developmental delay was associated with any soiling but not with constipation alone. Constipation alone may be more strongly related to family history, early experiences of painful defecation and defecation anxiety while soiling is related to delays in reaching social developmental milestones. We found no evidence that length of gestation or birthweight were associated with constipation or soiling at school age consistent with an earlier study.[19] After adjusting for confounders, earlier or later initiation of toilet training had no significant effect on constipation or soiling similar to previous studies[19,20] and in contrast to a large cohort study.[7]

This study finds evidence that experiencing hard stools in early childhood is a risk factor for later problems with constipation at school age. Early identification of children at risk of constipation is important because more than a third of cases become chronic and require secondary care.[30] Early diagnosis and treatment will reduce the risk of constipation persisting to school age resulting in poor quality of life. The burden of constipation is large - 13·2% of primary school age children. Extra health care costs are substantial as 5·9% of the ALSPAC cohort 4-9 years of age, saw a doctor at least once (data not shown). Many would have been referred to secondary

Confidential: For Review Only

care and tertiary paediatric gastroenterology and paediatric surgery services.

Further population-based studies are needed and if non-retentive soiling rates are confirmed, parents should be encouraged to seek help for soiling from clinicians who recognise the problem. As with daytime wetting the mainstay of treatment for soiling (alone and the soiling aspect of soiling with constipation) will be behavioural, for example supporting children to attempt to empty their bowels into the toilet at least once per day. This form of therapy requires practitioners to be skilled at providing behavioural interventions concurrently with medications for children who are constipated.

Confidential: For Review Only

Acknowledgements:

We are extremely grateful to all the families who took part in this study, the midwives for their help in recruiting them, and the whole ALSPAC team, which includes interviewers, computer and laboratory technicians, clerical workers, research scientists, volunteers, managers, receptionists and nurses.

Funding statement

The MRC and Wellcome Trust (Grant ref: 102215/2/13/2) and University of Bristol provide core support for ALSPAC. This publication is the work of the authors who will serve as guarantors for the contents of this paper. This work was supported by the MRC (MR/L007231/1) and NHS Greater Glasgow & Clyde Health Board (*Stephen McLeod* - Head of Specialist Children's Services).

Competing Interests statement:

The authors have no financial or personal relationships to declare.

Contributorship statement:

Secured funding: DT and CJ

Study design: JH, DT, AvG and CJ

Literature search: CJ

Data analysis: JH and MG

Data interpretation: JH, MG, DT, AvG and CJ

Writing: JH, MG, DT, AvG and CJ

Confidential: For Review Only

References

1. Kovacic K, Sood MR, Mugie S, et al. A multicenter study on childhood constipation and fecal incontinence: effects on quality of life. *J Pediatr* 2015; **166**: 1482–7.e1.
2. Bongers ME, Tabbers MM, Benninga MA. Functional nonretentive fecal incontinence in children. *J Pediatr Gastroenterol Nutr* 2007; **44**: 5–13. Review.
3. Hyams JS, Di Lorenzo C, Saps M, Shulman RJ, Staiano A, van Tilburg M. Functional Disorders: Children and Adolescents. *Gastroenterology* 2016: S0016–5085(16)00181–5.
4. Zeevenhooven J, Koppen IJN, Benninga MA. The New Rome IV Criteria for Functional Gastrointestinal Disorders in Infants and Toddlers. *Pediatric Gastroenterology, Hepatology & Nutrition* 2017; **20**: 1–13.
5. van den Berg MM, Benninga MA, Di Lorenzo C. Epidemiology of childhood constipation: a systematic review. *Am J Gastroenterol* 2006; **101**: 2401–9.
6. Mugie SM, Benninga MA, Di Lorenzo C. Epidemiology of constipation in children and adults: a systematic review. *Best Pract Res Clin Gastroenterol* 2011; **25**: 3–18
7. Bellman M. Studies on encopresis. *Acta Paediatr Scand* 1966; **170**: 1– 151
8. van der Wal MF, Benninga MA, Hirasing RA. The prevalence of encopresis in a multicultural population. *J Pediatr Gastroenterol Nutr* 2005; **40**: 345–8.
9. Chung JM, Lee SD, Kang DI, et al; An epidemiologic study of voiding and bowel habits in Korean children: a nationwide multicenter study. *Urology* 2010; **76**: 215–9.
10. Rajindrajith S, Devanarayana NM, Benninga MA. Constipation–associated and nonretentive fecal incontinence in children and adolescents: an epidemiological survey in Sri Lanka. *J Pediatr Gastroenterol Nutr* 2010; **51**: 472–6.

Confidential: For Review Only

11. Loening–Baucke V. Constipation in early childhood: patient characteristics, treatment, and longterm follow up. *Gut* 1993; **34**: 1400–4.

12. Partin JC, Hamill SK, Fischel JE, Partin JS. Painful defecation and fecal soiling in children. *Pediatrics* 1992; **89**: 1007–9.

13. Blum NJ, Taubman B, Osborne ML. Behavioral characteristics of children with stool toileting refusal. *Pediatrics* 1997; **99**: 50–3.

14. Weaver LT, Ewing G, Taylor LC. The bowel habit of milk–fed infants. *J Pediatr Gastroenterol Nutr* 1988; **7**: 568–71.

15. Park M, Bang YG, Cho KY. Risk Factors for Functional Constipation in Young Children Attending Daycare Centers. *J Korean Med Sci* 2016; **31**: 1262–5.

16. Ludvigsson JF1; Abis Study Group. Epidemiological study of constipation and other gastrointestinal symptoms in 8000 children. *Acta Paediatr* 2006; **95**: 573–80.

17. Hack M, Taylor HG, Klein N, Mercuri-Minich N. Functional limitations and special health care needs of 10– to 14–year–old children weighing less than 750 grams at birth. *Pediatrics* 2000; **106**: 554–60.

18. Chaidez V1, Hansen RL, Hertz–Picciotto I. Gastrointestinal problems in children with autism, developmental delays or typical development. *J Autism Dev Disord* 2014; **44**: 1117–27.

19. Largo RH, Molinari L, von Siebenthal K, Wolfensberger U. Development of bladder and bowel control: significance of prematurity, perinatal risk factors, psychomotor development and gender. *Eur J Pediatr* 1999; **158**: 115–22.

20. Blum NJ, Taubman B, Nemeth N. Relationship between age at initiation of toilet training and duration of training: a prospective study. *Pediatrics* 2003; **111**: 810–4.

Confidential: For Review Only

21. Schum TR, Kolb TM, McAuliffe TL, Simms MD, Underhill RL, Lewis M. Sequential acquisition of toilet–training skills: a descriptive study of gender and age differences in normal children. *Pediatrics* 2002; **109**: E48

22. Heron J, Joinson C, Croudace T, von Gontard A. Trajectories of daytime wetting and soiling in a United Kingdom 4 to 9–year–old population birth cohort study. *J Urol* 2008; **179**: 1970–5.

23. Boyd A, Golding J, Macleod J, et al. Cohort Profile: the 'children of the 90s'–the index offspring of the Avon Longitudinal Study of Parents and Children. *Int J Epidemiol* 2013; **42**: 111–27.

24. Frankenburg WK, Dodds J, Archer P, Shapiro H, Bresnick B. The Denver II: a major revision and restandardization of the Denver Developmental Screening Test. *Pediatrics* 1992; **89**: 91–97.

25. Muthén LK, Muthén BO. Mplus User's Guide. 7th Edition: Los Angeles, California: Muthén & Muthén, 2012.

26. Vermunt, J. K. Latent Class Modeling with Covariates: Two Improved Three–Step Approaches. *Political Analysis* 2010; **18**: 450–469.

27. Heron JE, Croudace TJ, Barker ED, Tilling K. A comparison of approaches for assessing covariate effects in latent class analysis. *Longitudinal and Life Course Studies* 2015; **6**: 420–434

28. Benninga MA, Büller HA, Heymans HS, Tytgat GN, Taminiau JA. Is encopresis always the result of constipation? *Arch Dis Child* 1994; **71**: 186–93.

29. Chan AO, Hui WM, Lam KF et al. Familial aggregation in constipated subjects in a tertiary referral center. *Am J Gastroenterol* 2007; **102**: 149–52.

Confidential: For Review Only

30. National Institute for Health and Care Excellence. Constipation in children and young people. https://www.nice.org.uk/guidance/qs62. Date accessed: 19/0917

Confidential: For Review Only

Figure 1. Trajectories for constipation and soiling

Confidential: For Review Only

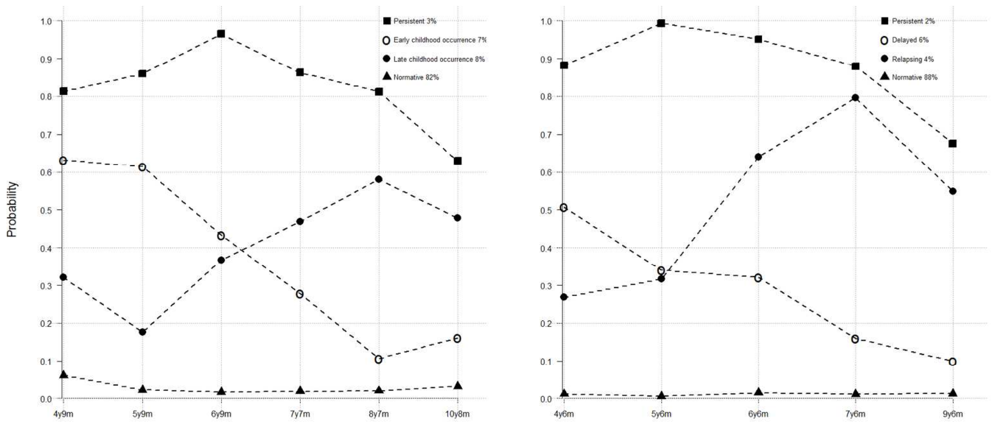

677x339mm (72 x 72 DPI)

For Review Only

Table S1. Goodness of fit statistics for LLCA model of constipation

Sample	# classes	SSA-BIC	Entropy	LMR-p	BLRT-p
Cases with complete data for six measures of constipation (N=5,435)	1 class	21959.6	-	-	-
	2 classes	18334.3	0.878	<0.001	<0.001
	3 classes	18239.7	0.836	<0.001	<0.001
	4 classes	18209.0	0.842	<0.001	<0.001
	5 classes	18229.5	0.863	0.223	0.090
Cases with at least 3 non-missing measures of constipation (N=8,979)	1 class	32429.9	-	-	-
	2 classes	27293.9	0.859	<0.001	<0.001
	3 classes	27159.6	0.808	<0.001	<0.001
	4 classes	27078.8	0.818	<0.001	<0.001
	5 classes	27103.7	0.836	0.661	0.050
Cases with at least 1 non-missing measure of constipation (N=11,195)	1 class	34855.7	-	-	-
	2 classes	29567.9	0.813	<0.001	<0.001
	3 classes	29431.7	0.772	<0.001	<0.001
	4 classes	29342.3	0.785	<0.001	<0.001
	5 classes	29367.8	0.805	0.600	0.050

The LLCA model permits the inclusion of partial respondents (those who provide some but not all repeated measures) through the use of Full Information Maximum Likelihood (FIML) estimation. FIML is a commonly used approach for estimating models within the SEM framework and is often computationally less demanding than Multiple Imputation (MI). Both FIML and MI are based on the Missing At Random assumption. In the case of FIML, MAR equates to the notion that, conditional on the variables in the estimated model, there are no systematic differences between observed and non-observed values of any partially complete variable.

SSA-BIC - sample-size adjusted Bayesian Information Criterion. The Bayesian Information Criterion (BIC) is the traditional fit statistic for comparing mixture models. BIC will typically decrease and then increase following the incremental additional of classes. Using this statistic, the model with the lowest BIC (or other models with BIC values in that vicinity) would be deemed optimal. There is good support for 4-classes using the BIC.

Entropy. This is a measure of classification accuracy, and whilst it is generally of little use in determining the optimal model, it indicates the level of bias which one would expect were a standard three-step estimation to be performed. Here entropy is fairly high and above the commonly-used threshold of 0.8 for all models. We have recently demonstrated (Heron, Croudace, Barker, & Tilling, 2015) that bias can result even for values reaching 0.9 and hence we have opted for the bias-adjusted three-step method as described in the methods.

The *Bootstrap Likelihood Ratio Test (BLRT)* and the *Lo-Mendell-Rubin (LMR)* test statistics both assess change in model fit when adding an additional class. Here a high p-value for a k-class model indicates no substantial improvement in fit compared to the k-1 class solution. Whilst the BLRT is usually more conservative, both are in support of a 4-class solution.

Heron, J. E., Croudace, T. J., Barker, E. D., & Tilling, K. (2015). A comparison of approaches for assessing covariate effects in latent class analysis. Longitudinal and Life Course Studies; Vol 6, No 4 (2015): Longitudinal and Life Course Studies.

Confidential: For Review Only

Table S2. Goodness of fit statistics of parallel LLCA models including between 3 and 5 classes both on soiling and constipation dimension.

# soiling classes	# constipation classes	Complete data for both soiling *and* constipation (N=4,931)		At least 3 measures each for both soiling *and* constipation (N=8,435)		At least 1 measure each for both soiling *and* constipation (N=10,450)	
		Entropy	SSA-BIC	Entropy	SSA-BIC	Entropy	SSA-BIC
3	3	0.833	25598.3	0.823	40364.7	0.801	44632.6
3	4	0.843	25574.4	0.832	40298.0	0.810	44563.6
3	5	0.847	25602.4	0.846	40328.6	0.826	44596.9
4	3	0.859	25594.6	0.846	40360.2	0.825	44628.7
4	4	0.879	**25571.9**	0.852	**40285.3**	0.830	**44552.1**
4	5	0.882	25603.4	0.866	40317.3	0.842	44586.0
5	3	0.841	25620.5	0.834	40393.9	0.794	44664.7
5	4	0.884	25597.3	0.870	40321.8	0.850	44589.9
5	5	0.893	25631.1	0.884	40357.0	0.861	44627.6

There was good support for a 4-class solution for both constipation and soiling when considered separately. When both sets of data were brought together for the estimation of a joint model we considered 3-, 4- and 5-class solutions for both dimensions. The reason being that the additional information can distort the estimation of the two latent class measurement models, particularly when missing data is present. In the current situation, there remained good support for a 4-class by 4-class solution and the individual trajectories estimated for soiling and constipation were not adversely affected.

Confidential: For Review Only

Table S3. Association between risk factors and the latent classes of (i) Constipation, and (ii) Soiling

	(i) Constipation classes (n ≤ 8,979)				(ii) Soiling classes (n ≤ 8,764)			
	Early Childhood Occurrence OR [95% CI]	Late Childhood Occurrence OR [95% CI]	Persistent OR [95% CI]	Omnibus p-value	Delayed OR [95% CI]	Relapse OR [95% CI]	Persistent OR [95% CI]	Omnibus p-value
Hard stools regularity at 2.5 yrs				<0.001				0.146
Never hard (ref.)	1.00 ref				1.00 ref			
Sometimes	2.28 [1.32, 3.92]	1.47 [0.99, 2.20]	1.88 [1.11, 3.18]		1.00 [0.55, 1.81]	1.27 [0.75, 2.15]	0.79 [0.52, 1.19]	
Usually hard	2.24 [1.26, 3.98]	1.30 [0.83, 2.03]	2.19 [1.27, 3.78]		0.84 [0.43, 1.64]	0.74 [0.39, 1.40]	0.73 [0.46, 1.17]	
Breastfeeding by 6 months				0.121				0.588
child never breastfed (ref.)	1.00 ref				1.00 ref			
child breastfed for < 6mn	1.03 [0.69, 1.54]	1.46 [0.98, 2.16]	1.05 [0.71, 1.55]		1.30 [0.73, 2.32]	1.52 [0.87, 2.68]	0.85 [0.56, 1.28]	
child breastfed for at least 6mn	1.35 [0.90, 2.00]	1.39 [0.92, 2.10]	0.75 [0.48, 1.19]		1.12 [0.60, 2.09]	1.30 [0.71, 2.38]	0.99 [0.65, 1.50]	
Sex				<.001				<.001
Female (ref.)	1.00 ref				1.00 ref			
Male	0.81 [0.61 1.07]	0.64 [0.49, 0.84]	0.84 [0.62, 1.13]		1.45 [0.95, 2.22]	1.91 [1.30, 2.80]	2.00 [1.42, 2.83]	
Parental social class				0.066				0.721
Non-manual (ref)	1.00 ref				1.00 ref			
Manual	0.81 [0.52, 1.25]	0.68 [0.44, 1.05]	1.43 [0.98, 2.07]		0.76 [0.38, 1.55]	1.19 [0.74, 1.91]	1.16 [0.74, 1.80]	
Maternal educational attainment				0.121				0.795
A-level/degree (ref.)	1.00 ref				1.00 ref			
O-level	0.84 [0.60, 1.17]	0.88 [0.65, 1.18]	1.32 [0.93, 1.89]		1.07 [0.67, 1.70]	0.84 [0.55, 1.30]	0.91 [0.62, 1.32]	
< O-level	0.82 [0.57, 1.19]	0.64 [0.44, 0.94]	1.22 [0.82, 1.82]		0.67 [0.35, 1.28]	1.07 [0.69, 1.66]	1.00 [0.66, 1.51]	
Material hardship at 33 months				0.144				0.087
no hardship [score<5] (ref.)	1.00 ref				1.00 ref			
material hardship [score ≥ 5]	1.18 [0.86, 1.63]	1.00 [0.73, 1.37]	1.38 [0.99, 1.92]		0.83 [0.48, 1.42]	1.11 [0.74, 1.68]	1.53 [1.07, 2.18]	

Confidential: For Review Only

Table S3. Continued

	(i) Constipation class				(ii) Soiling class			
	Early Childhood Occurrence OR [95% CI]	Late Childhood Occurrence OR [95% CI]	Persistent OR [95% CI]	Omnibus p-value	Delayed OR [95% CI]	Relapse OR [95% CI]	Persistent OR [95% CI]	Omnibus p-value
Home ownership at 33 months				0.022				0.791
Home owned/mortgaged (ref.)	1.00 ref				1.00 ref			
Privately rented	0.89 [0.42, 1.86]	0.58 [0.24, 1.41]	0.97 [0.42, 2.21]		1.05 [0.35, 3.16]	1.27 [0.56, 2.88]	0.89 [0.34, 2.35]	
Subsidised rented	0.77 [0.46, 1.28]	0.47 [0.25, 0.90]	1.73 [1.17, 2.57]		0.88 [0.43, 1.80]	0.56 [0.24, 1.28]	1.14 [0.69,1.88]	
Car ownership at 33 months				0.510				0.057
yes, car owned (ref.)	1.00 ref				1.00 ref			
no car	1.28 [0.79, 2.07]	0.65 [0.33, 1.26]	1.16 [0.67, 2.01]		1.32 [0.63, 2.77]	0.89 [0.40, 1.97]	1.90 [1.16, 3.09]	
Gestation age at delivery				0.248				0.309
≥ 37weeks (ref.)	1.00 ref				1.00 ref			
< 37 weeks	0.97 [0.84, 1.11]	0.49 [0.21, 1.14]	0.79 [0.39, 1.60]		1.49 [0.67, 3.27]	1.12 [0.50, 2.51]	1.50 [0.81, 2.77]	
Birth weight				0.075				0.467
≥ 2500g (ref.)	1.00 ref				1.00 ref			
< 2500g	1.13 [0.84, 1.53]	0.84 [0.72, 0.99]	1.38 [0.80, 2.37]		0.73 [0.21, 2.60]	1.23 [0.56, 2.68]	1.56 [0.83, 2.94]	
Developmental level at 18m				0.189	1.00 ref			<0.001
Per 1 SD reduction in development	0.97 [0.81, 1.18]	0.91 [0.77, 1.07]	1.21 [1.01, 1.46]		1.27 [1.04, 1.54]	1.31 [1.05, 1.63]	1.66 [1.39, 2.00]	
Toilet training initiation				0.991				0.012
Before 6mn	1.31 [0.52, 3.29]	1.16 [0.47, 2.85]	1.21 [0.44, 3.35]		1.91 [0.54, 6.78]	2.01 [0.78, 5.15]	1.13 [0.30, 4.30]	
Between 6 &15mn	1.01 [0.63, 1.59]	1.10 [0.74, 1.64]	1.00 [0.61, 1.62]		1.26 [0.66, 2.42]	0.70 [0.35, 1.41]	0.99 [0.56, 1.74]	
Started between 15 & 24mn (ref.)	1.00 ref				1.00 ref			
Started after 24mn	1.13 [0.82, 1.55]	0.90 [0.66, 1.24]	0.99 [0.70, 1.41]		1.60 [0.99, 2.58]	1.15 [0.76, 1.74]	1.62 [1.12, 2.33]	

Confidential: For Review Only

Figure S1. Derivation of samples used for analysis

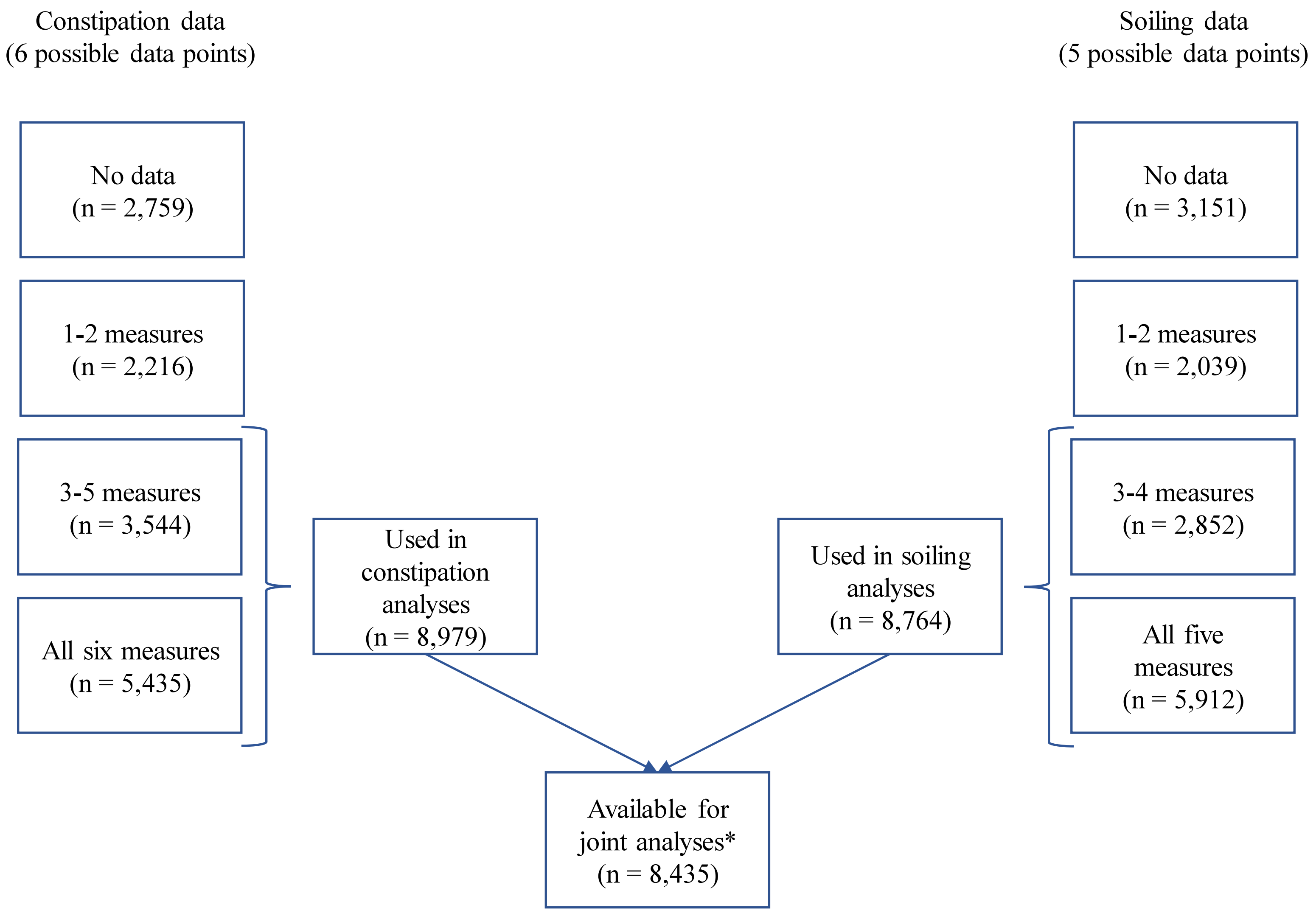

* Joint analysis focussed on the sample with 3+ measures on both constipation and soiling

5

Figure S2. Rates of daytime wetting, bedwetting, stomach ache and infrequent bowel movements within classes of children defined by their constipation and daytime soiling in mid-childhood.

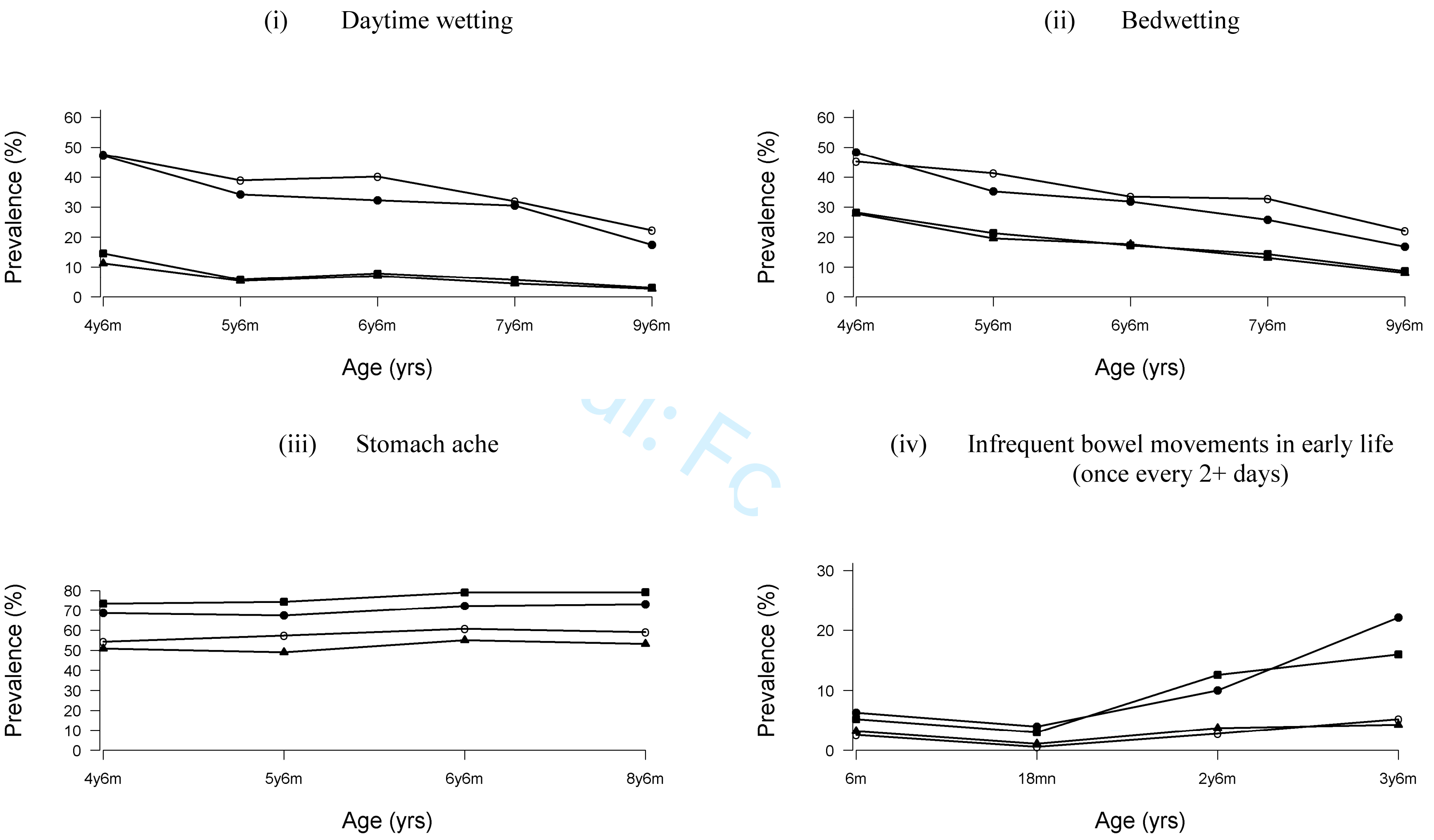

Constipation with soiling (●), Soiling alone (○), Constipation alone (■), Normative (▲)
